# Numerical modeling of the funnel multiphysical flow of fresh self-compacting concrete considering proportionate heterogeneity of aggregates

**DOI:** 10.1038/s41598-024-52072-w

**Published:** 2024-01-18

**Authors:** Kennedy C. Onyelowe, Denise-Penelope N. Kontoni

**Affiliations:** 1 Department of Civil Engineering, School of Engineering, University of the Peloponnese, 26334 Patras, Greece; 2 Department of Civil Engineering, Michael Okpara University of Agriculture, Umudike, Nigeria; 3 School of Science and Technology, Hellenic Open University, 26335 Patras, Greece

**Keywords:** Engineering, Materials science

## Abstract

Filling ability is one of the prominent rheological properties of the self-compacting concrete (SCC), which has been studied in this research work deploying the functional behavior of the concrete through the studied funnel apparatus using the coupled ANSYS-SPH interface. Seven (7) model cases were studied and optimized. The aim of this numerical study is to propose a more sustainable mix of coarse and fine aggregates proportion that allows for most minimum flow time to enhance a more efficient filling of forms during concreting. The maximum size of the coarse aggregates considered is 20 mm and that of the fine aggregates is below 4 mm. The Bingham model properties for the multiphysics (SPH)-ANSYS models’ simulation are; viscosity = 20 ≤ μ ≤ 100 and the yield stress = 50 $\le {\tau }_{0}\le 200$, standard flow time, t (s) ranges; 6 ≤ t ≤ 25 and the funnel volume is 12 L. The minimum boundary flow time, which represents the time it takes for the SCC to completely flow through a specified distance, typically measured in seconds was modeled for in the seven (7) model cases. The second case with 40% coarse mixed with 60% fine completely flowed out in 16 s, thus fulfilling the minimum flow time. This minimum flow time was considered alongside other relevant parameters and tests, such as slump flow, passing ability, segregation resistance, and rheological properties (stresses), to comprehensively assess the filling ability of SCC in this model. By considering these factors and the optimized mix (40%C + 60%F:16s), engineers and researchers can optimize the SCC mix design to achieve the desired flowability and filling performance for their specific construction applications. The multiphase optimized mix was further simulated using the coupled interface of the ANSYS-SPH platform operating with the CFX command at air temperature of 25 °C. The results show energy reduction jump at the optimized flow time. Ideally, the mix, 40%C + 60%F:16s has been proposed as the mix with the most efficient flow to achieve the filling ability for sustainable structural concrete construction.

## Introduction

The multiphysical flow of fresh self-compacting concrete (SCC) takes into account the proportionate heterogeneity of fine and coarse aggregates^1^. SCC is a specialized type of concrete that is highly workable and able to flow and fill complex forms under its own weight without the need for mechanical consolidation^2^. When evaluating the funnel flowability of fresh self-compacting concrete (SCC) while considering the heterogeneity of aggregates, you may need to account for the variation in particle sizes and shapes within the mixture^1^. Approaches that can help incorporate aggregate heterogeneity into the V-funnel flowability assessment include^1^: (a) Aggregate characterization: by characterizing the aggregates in the SCC mixture^2,3^. Obtain information about the particle size distribution, shape, and surface texture of the aggregates. This data can be obtained through sieve analysis, imaging techniques, or other suitable methods^2,4^. (b) Aggregate modeling: develop a representative aggregate model that captures the heterogeneity of the aggregates^1,2^. This can involve creating a discrete representation of the aggregate particles or using statistical distributions to represent their properties (e.g., size, shape)^5^. (c) Mix design: perform a mix design for SCC, considering the aggregate characteristics^1^. Adjust the proportion of aggregates and other constituents (cement, water, admixtures) to achieve the desired flowability and workability^5–7^. (d) Numerical simulation: Utilize numerical simulation techniques, such as the Discrete Element Method (DEM), to simulate the flow behavior of SCC in the V-funnel^2–8^. DEM allows for the individual representation of aggregates as discrete particles, accounting for their size, shape, and interactions^6^. (e) Model calibration: calibrate the numerical model by comparing the simulation results with experimental measurements of V-funnel flowability^9^. Adjust the parameters of the aggregate model (e.g., particle size distribution, shape parameters) to achieve a good agreement between the simulated and measured flowability values^10^. (f) Sensitivity analysis: perform sensitivity analyses to investigate the influence of different aggregate properties (e.g., particle size distribution, shape) on the V-funnel flowability^9,10^. This can help you understand how variations in aggregate heterogeneity affect the flow behavior of SCC. By incorporating aggregate heterogeneity into the numerical simulation of V-funnel flowability, you can gain insights into the impact of aggregates on the workability and flow characteristics of SCC^1,11^. This information can be valuable for optimizing mix designs and improving the performance of self-compacting concrete in practical applications^4^. It’s important to note that the numerical simulation approach described above requires expertise in numerical modeling and an understanding of the specific software tools available for DEM simulations^6–8^. Additionally, experimental validation and further analysis may be necessary to fully evaluate the impact of aggregate heterogeneity on SCC flowability.

When considering the numerical modeling of V-funnel flowability of fresh self-compacting concrete (SCC) while incorporating the heterogeneity of aggregates, coupling ANSYS with SPH can be a challenging task due to the different numerical methods employed by each software^2–8^. However, I can provide a general strategy that combines the two methods to simulate the flow behavior of SCC with heterogeneous aggregates in the V-funnel: (a) preprocessing: create the geometry of the V-funnel and define the boundary conditions in ANSYS^11–14^. Define the material properties and constitutive models for the SCC mixture in ANSYS. Generate the SPH particles that represent the SCC mixture with heterogeneous aggregates using an SPH software package^3,15–17^. Assign properties to the particles based on the aggregate characteristics. (b) Data exchange: develop a data exchange interface between ANSYS and the SPH solver. This interface should allow for the transfer of relevant information, such as particle positions, velocities, and material properties^8^. Transfer the initial geometry, boundary conditions, and material properties from ANSYS to the SPH solver. (c) Simulation: a. Perform the SPH simulation using the transferred data. The SPH solver will simulate the flow behavior of the SCC mixture, accounting for the heterogeneity of aggregates^16^. b. Extract the relevant simulation results from the SPH solver, such as velocity and pressure distributions. (d) Postprocessing: a. Transfer the simulation results back from the SPH solver to ANSYS^17^. b. Analyze and visualize the results in ANSYS to obtain insights into the V-funnel flowability of the SCC with heterogeneous aggregates^3^. c. Perform further postprocessing tasks, such as computing flow rates or assessing the effect of aggregate heterogeneity on the flow behavior. Also, note that the coupling process between ANSYS and SPH may require custom development of data exchange interfaces and scripts, as well as a deep understanding of both software packages^18^. Additionally, the numerical parameters and modeling assumptions in both ANSYS and SPH need to be carefully selected and validated to ensure accurate and reliable results^2,8,19^. Also, the specific implementation details may vary depending on the versions of ANSYS and the SPH software you are using, as well as the specific requirements of your simulation^20^. It’s recommended to consult the documentation and resources specific to your software versions or seek assistance from experts in ANSYS and SPH software for detailed instructions and support^1^.

The constitutive equations for v-funnel flowability of fresh self-compacting concrete (SCC) considering the heterogeneity of aggregates in a coupled ANSYS-SPH simulation would depend on the specific constitutive models used for the SCC and the numerical methods employed by ANSYS and SPH^20^. The constitutive model for SCC typically includes the following equations:- Bingham Plastic Model: The Bingham model can describe the behavior of SCC under shear stress^1,21^. It assumes that the material behaves as a rigid body until a yield stress is exceeded, after which it flows like a viscous fluid^22^. Rheological Models: More sophisticated rheological models such as the Herschel-Bulkley model or the Cross model can also be used to capture the non-linear flow behavior of SCC^23^. These models consider the yield stress, plastic viscosity, and other parameters to represent the flow characteristics accurately^22^. (b) ANSYS provides a range of material models and constitutive equations that can be applied to simulate the behavior of concrete^19^. The specific constitutive equations in ANSYS would depend on the model chosen, such as the Bingham Plastic Model or other rheological models^20^. (c) In the SPH simulation, the constitutive equations typically involve the following aspects: Particle Interactions: SPH uses a kernel function to describe the interactions between particles^23^. The kernel function determines the particle–particle forces, such as pressure and viscosity forces, based on the particle properties and their relative positions^24^. Material Behavior: The constitutive equations in SPH can be based on continuum mechanics principles, such as the Navier–Stokes equations, which describe the conservation of mass and momentum^25^. These equations can incorporate the material properties, such as density, viscosity, and yield stress, to simulate the flow behavior of SCC^26^. (d) The coupling between ANSYS and SPH involves the exchange of relevant information, such as particle positions, velocities, and material properties^21^. The constitutive equations governing the flow behavior of SCC are typically implemented within each software package separately, and the coupling focuses on data exchange and synchronization between the two solvers^21^. It’s important to note that the specific constitutive equations used for SCC and the numerical methods employed by ANSYS and SPH may vary depending on the software versions, simulation requirements, and modeling assumptions^22^. It’s recommended to consult the documentation and resources specific to the ANSYS and SPH software versions you are using, as well as the relevant literature on SCC modeling, for detailed information on the constitutive equations and their implementation in your simulation setup^23^.

The v-funnel test is commonly used to assess the flowability or workability of fresh self-compacting concrete (SCC)^24^. The test measures the time it takes for the concrete to flow through a standardized funnel. The continuity equation for v-funnel flowability considers the heterogeneity of aggregates in the following manner: (a) Volume Continuity Equation: The volume continuity equation relates the flow rate of concrete through the v-funnel to the cross-sectional area and the velocity of flow^3^. It can be expressed as follows: Q = A × v. Where: Q is the flow rate of concrete (m^3^/s), A is the cross-sectional area of the v-funnel (m^2^), v is the average velocity of concrete flow (m/s)^2^. (b) Velocity Profile Equation: The velocity profile equation describes the variation in the velocity of concrete flow across the cross-section of the v-funnel^7^. In the case of SCC with heterogeneous aggregates, the velocity profile can be assumed to be non-uniform^25^. This can be represented by a velocity coefficient, which varies along the radial direction of the funnel: v = V × C. Where: v is the local velocity of concrete flow (m/s), V is the average velocity of concrete flow (m/s), C is the velocity coefficient. (c). Aggregate Heterogeneity: In SCC, the heterogeneity of aggregates can affect the flowability of concrete. The presence of different aggregate sizes and shapes can influence the velocity coefficient C^26^. The velocity coefficient can be considered as a function of the radial position within the v-funnel, denoted as r: C = f(r). The specific form of the function f(r) would depend on the aggregate distribution and properties^5^. To determine the continuity equations for a specific case of SCC with heterogeneous aggregates, it would be necessary to conduct experimental tests and analyze the flow behavior. These equations can then be derived based on the observed flow patterns and properties of the concrete^2–8^.

The Navier–Stokes equations are a set of partial differential equations that describe the motion of fluid substances, including liquids like fresh self-compacting concrete (SCC)^6^. These equations can be used to analyze the flow behavior of SCC in a v-funnel, taking into account the heterogeneity of aggregates^9^. However, it should be noted that solving the full Navier–Stokes equations for complex flow situations like v-funnel flowability of SCC can be challenging and often requires simplifications and assumptions^4^. In the case of SCC flow through a v-funnel, the Navier–Stokes equations can be written as follows: (a). Continuity Equation: ∇ · (ρv) = 0. This equation represents the conservation of mass, where ρ is the density of the SCC and v is the velocity vector. (b). Momentum Equation: ρ (∂v/∂t + v · ∇v) = − ∇P + μ∇^2^v + ρg. This equation represents the conservation of momentum, where P is the pressure, μ is the dynamic viscosity of the SCC, g is the gravitational acceleration vector, and ∇^2^v represents the Laplacian of the velocity vector. To incorporate the heterogeneity of aggregates, the Dirac delta function can be used to represent the concentration or distribution of aggregates within the SCC^2^. The Dirac delta function, denoted as δ(r), is a mathematical function that is zero everywhere except at the origin, where it is infinite, and its integral over any region containing the origin is equal to 1^3^. For example, if you want to represent the effect of aggregates on the density, velocity, or viscosity of the SCC, you can introduce the Dirac delta function as a spatial distribution function. The specific form of this distribution function would depend on the nature of the aggregates and their spatial distribution within the SCC^4^. However, it’s important to note that the application of Navier–Stokes equations and the Dirac delta function to model the v-funnel flowability of SCC with aggregate heterogeneity requires simplifying assumptions and empirical correlations^22^. The complexity and non-linearity of the flow behavior make it challenging to obtain analytical solutions, and numerical methods or experimental investigations are often employed to analyze and predict the flow properties of SCC in v-funnel tests^16^.

The Bingham plastic model is commonly used to describe the flow behavior of materials that exhibit a yield stress, such as fresh self-compacting concrete (SCC)^11^. The model assumes that the material behaves as a solid until a certain stress threshold, known as the yield stress, is exceeded^13^. Once the yield stress is surpassed, the material flows like a viscous fluid^14^. To incorporate the heterogeneity of aggregates in the v-funnel flowability of SCC, the Bingham plastic model can be modified as follows: (a) Yield Stress: The yield stress τ_y is the minimum stress required to initiate flow^21^. In the case of SCC with aggregate heterogeneity, the yield stress can vary spatially^12^. The yield stress can be represented as a function of position within the v-funnel, denoted as τ_y(r):

τ_y = f(r). The specific form of the function f(r) would depend on the aggregate distribution and properties. (b). Flow Behavior: Once the yield stress is exceeded, the SCC flows like a viscous fluid. The velocity profile can be described using the Bingham equation^11^: τ = τ_y + μ ∂v/∂z. Where: τ is the shear stress (Pa), μ is the dynamic viscosity of the SCC (Pa·s), ∂v/∂z is the velocity gradient in the direction of flow. (c). Continuity Equation: The continuity equation for the v-funnel flowability of SCC, considering the Bingham plastic model, relates the flow rate of concrete to the cross-sectional area and the velocity of flow^22–26^. It can be expressed as: Q = A × ∫[τ(r)]/μ dz. Where: Q is the flow rate of concrete (m^3^/s), A is the cross-sectional area of the v-funnel (m^2^), ∫[τ(r)]/μ dz represents the integral of the shear stress distribution over the height of the v-funnel. To determine the specific equations for a given case of SCC with heterogeneous aggregates, it would be necessary to conduct experimental tests and analyze the flow behavior^16,21^. These equations can then be derived based on the observed flow patterns and properties of the concrete.

To model the v-funnel flowability of fresh self-compacting concrete (SCC) considering particle interaction and coupling between ANSYS and Smoothed Particle Hydrodynamics (SPH) equations, the following steps can be followed^11–16^: (a). Particle Interaction Modeling: a. Define the discrete particles representing the SCC in the ANSYS software^21^. Each particle should have properties such as mass, position, velocity, and size. b. Use appropriate contact models to simulate the interaction between particles, considering the heterogeneity of aggregates^15^. The contact models should account for particle–particle interactions, including collisions, cohesion, and friction. (b). Smoothed Particle Hydrodynamics (SPH) Modeling: a. Implement the SPH method, which is a meshless Lagrangian particle-based method, to solve the fluid flow equations for the SCC. b. Define the SPH equations for fluid flow, including the continuity equation and the Navier–Stokes equations^21^. These equations describe the conservation of mass and momentum in the SCC^24^. c. Incorporate the Bingham plastic model to account for the SCC’s yield stress and rheological behavior when the stress exceeds the yield stress. (c). Coupling ANSYS and SPH: a. Establish the coupling between the ANSYS particle model and the SPH fluid flow model^12^. This involves exchanging information between the two models at each time step. b. Update the positions and velocities of the discrete particles in the ANSYS model based on the fluid flow solution obtained from the SPH equations. c. Recalculate the particle–particle interactions in the ANSYS model using the updated positions and velocities^13^. (d). Heterogeneity of Aggregates: a. Introduce the heterogeneity of aggregates into the particle model by assigning different properties, such as size, shape, and density, to individual particles based on the aggregate distribution^14^. b. Consider the spatial distribution of aggregates when defining the contact models and interaction forces between particles. c. Adjust the Bingham plastic model parameters, such as yield stress and viscosity, based on the properties of the aggregates^21^. It’s important to note that implementing such a coupled ANSYS-SPH approach for v-funnel flowability of SCC considering aggregate heterogeneity requires expertise in both software tools and an understanding of the specific properties and behavior of the SCC being modeled. It may also require validation against experimental data to ensure the accuracy of the simulation results.

## Theory and formulation

### Governing equations

**Figure 1:**
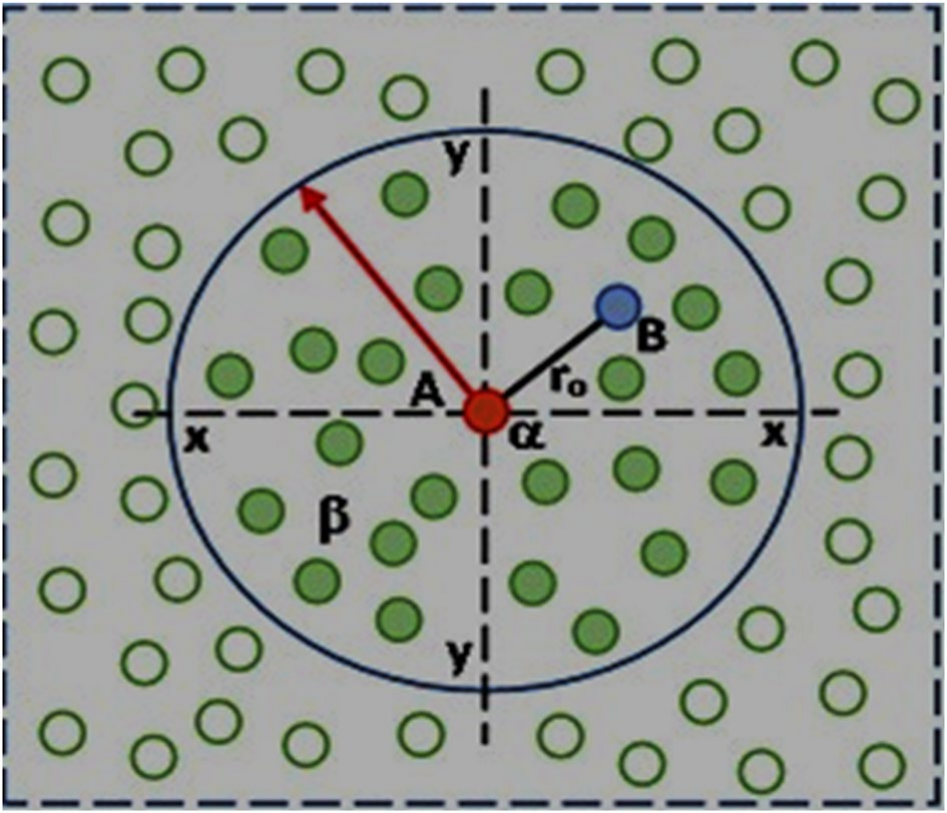
The SPH problem domain of continuous particles in SCC.

Smoothed Particle Hydrodynamics (SPH) serves as a computational technique utilized for simulating fluid dynamics and various physical phenomena^2–8^. This method finds widespread application in fields like astrophysics, computational fluid dynamics, and computer graphics. In SPH, a fluid is represented as a group of particles that interact with each other through a kernel function. State variables such as density, pressure, and velocity are associated with each particle^22–24^. To determine the values of these variables at any spatial point, a smoothing operation is executed using neighboring particles. The fundamental concept involves each particle contributing to the values of its neighbors through a kernel function, assigning weights to neighboring particles based on their distance from the target point^21–24^. Taking a scalar quantity like density as an example, the density at a specific spatial point is calculated by summing the contributions from nearby particles, as illustrated in Fig. 1.

The SPH density estimator is defined as:

1 $$\uprho \left( {\text{x}} \right) \, = \, \sum \left( {{\text{m}}_{{\text{j}}} *{\text{ W}}\left( {\left| {{\text{x }} - {\text{ x}}_{{\text{j}}} } \right|,{\text{ h}}} \right)} \right)$$

where ρ(x) is the density at point x, m_j_ is the mass of the jth particle, x_j_ is the position of the jth particle, W is the kernel function, |x − x_j_| is the distance between the target point x and the jth particle, h is the smoothing length that determines the range of influence of each particle.

The kernel function W(|x − xj|, h) assigns weights to neighboring particles based on their distance from x. Commonly employed kernel functions in SPH encompass the Gaussian kernel and the cubic spline kernel^22^. These functions adhere to specific properties, such as normalization and compact support, ensuring precision and stability in simulations^21^. Following the computation of density, other variables like pressure and velocity can be derived by solving relevant equations^16^. For instance, the pressure may be determined using an equation of state linking the fluid’s density and pressure^17^. Particle velocity can be updated by accounting for pressure and viscous forces at play^25^. Alongside density, various physical quantities are estimated using analogous SPH formulations^1^. The core concept involves computing these values by aggregating contributions from neighboring particles utilizing suitable kernel functions^23^. It’s noteworthy that SPH operates as a meshless method, indicating it doesn’t rely on a fixed grid or mesh to discretize the computational domain^11^. Instead, it dynamically adjusts the particle distribution based on fluid density and other properties^22,23,26^. The mathematical intricacies of SPH can become quite intricate, particularly when dealing with complex physical phenomena and their governing equations^11^. While the above explanation provides a simplified overview of SPH’s fundamental principles, researchers have developed more advanced techniques and refinements to enhance the accuracy and efficiency of SPH simulations^15^.

ANSYS and Smoothed Particle Hydrodynamics (SPH) are two different computational methods commonly used for solving engineering problems^23^. ANSYS is a commercial finite element analysis software suite that can handle a wide range of physics, including fluid flow, structural analysis, electromagnetics, and more^12^. On the other hand, SPH is a meshless Lagrangian method specifically designed for simulating fluid flows^5^. While ANSYS and SPH can be used independently, they can also be coupled together to take advantage of the strengths of both methods^25^. The coupling of ANSYS and SPH involves integrating the fluid flow simulations performed using SPH with the structural analysis capabilities of ANSYS^26^. The basic idea behind the coupling is to transfer information between the SPH solver and ANSYS^20^. This information can include forces and pressures acting on the fluid particles computed by the SPH solver, which are then used as inputs for the structural analysis in ANSYS^18^. Conversely, the deformation and motion of the solid structures in ANSYS can be used to update the boundary conditions for the SPH solver^20^. The specific equations used in the coupled ANSYS-SPH simulations depend on the physics being modeled^15^. In general, the fluid flow equations in SPH are based on the continuity equation and the Navier–Stokes equations, which are solved using particle-based methods^1^. The solid mechanics equations in ANSYS are typically based on the theory of elasticity or other appropriate constitutive models^9^. The coupling of ANSYS and SPH requires careful implementation and coordination between the two solvers^10^. It involves data transfer, interpolation, and synchronization between the fluid and structural domains. The process may require custom scripting or programming to establish the necessary communication protocols between the two software platforms^11^. It’s worth noting that the specific details of the coupled ANSYS-SPH equations, implementation, and workflow may vary depending on the specific software versions, available interfaces, and the problem being solved^12^. It is recommended to consult the software documentation, tutorials, and support resources provided by ANSYS and the SPH solver being used for more specific guidance on implementing a coupled ANSYS-SPH simulation. Solving the Navier–Stokes equations for Bingham fluid flow involves considering the behavior of a viscoplastic fluid that exhibits a yield stress^16^. The Navier–Stokes equations, which describe the conservation of momentum and mass for fluid flow, need to be modified to account for the Bingham fluid model^22^. The Bingham fluid model assumes that the fluid behaves like a solid until a certain stress level, called the yield stress, is exceeded. The modified Navier–Stokes equations for Bingham fluid flow can be written as follows: (a). Conservation of Mass: ∇ · (ρu) = 0. (b). Conservation of Momentum: ρ[∂u/∂t + (u · ∇)u] = − ∇p + ∇ · τ + ρg. where: ρ is the fluid density, u is the fluid velocity vector, t is time, p is the fluid pressure, τ is the deviatoric stress tensor, and g is the acceleration due to gravity. The deviatoric stress tensor, τ, for the Bingham fluid model is given by: τ = μ(Du − Dp), where: μ is the dynamic viscosity of the fluid, Du is the rate-of-deformation tensor, and Dp is the plastic deformation tensor. The plastic deformation tensor, Dp, is defined as: Dp = 0, for ||τ||≤ τy, and Dp = (1/2) * (||τ|| − τy) * (τ/||τ||), for ||τ||> τy, where: τy is the yield stress of the Bingham fluid and—||τ|| represents the magnitude of the deviatoric stress tensor τ^22^. To solve these equations numerically, appropriate boundary conditions and initial conditions must be defined^21^. Additionally, a suitable numerical method, such as finite difference, finite volume, or finite element, can be employed to discretize the equations in space and time^11^. The solution can then be obtained by iterating through time steps until convergence is achieved^12^. It is worth noting that solving the Navier–Stokes equations for Bingham fluid flow can be computationally demanding due to the presence of non-linear terms and the need to consider the plastic deformation tensor^15^. Therefore, efficient numerical methods and high-performance computing techniques may be necessary, particularly for complex flow geometries or large-scale simulations^19^.

The continuity equation describes the conservation of mass for fluid flow and is typically expressed as:

2 $$\nabla \cdot \, \left( {\uprho {\text{V}}} \right) \, + \, \partial\uprho /\partial {\text{t }} = \, 0$$

where ∇ is the divergence operator, ρ is the fluid density, V is the velocity vector, and ∂ρ/∂t is the rate of change of density with respect to time. For Bingham fluid flow, the continuity equation remains the same as the general form^22^. However, the velocity field and the density profile are modified to account for the behavior of Bingham fluids, which exhibit a yield stress and behave like solids until a certain stress threshold is exceeded^23^. To solve the continuity equation for Bingham fluid flow, you need to consider the following modifications: (a). Velocity field: The velocity field for Bingham fluid flow can be expressed as:

3 $${\text{V }} = {\text{ V}}_{{0}} + {\text{ V}}_{{1}}$$

where V_0_ is the plug flow velocity (uniform velocity within the fluid) and V_1_ is the shear rate-dependent velocity caused by the applied stress^21^. The magnitude of V_1_ varies depending on the local shear rate and the yield stress of the Bingham fluid. (b). Density profile: Bingham fluids typically have constant density within the fluid, with no significant density variations unless there are additional factors involved (e.g., temperature variations, chemical reactions). Hence, ρ can be considered as a constant unless there are specific reasons to include density variations. By incorporating these modifications into the continuity equation, you can solve for the Bingham fluid flow. However, it’s important to note that the specific approach and equations used to solve the problem may depend on the geometry, boundary conditions, and additional governing equations (e.g., momentum equations) that need to be considered.

To solve the Bingham plastic model equations for Bingham fluid flow, you need to consider the conservation of momentum equation (Navier–Stokes equation) along with the Bingham constitutive equation^22^. The Bingham constitutive equation describes the relationship between the shear stress and the velocity gradient in a Bingham fluid^24^. (a). Conservation of Momentum (Navier–Stokes Equation):

The Navier–Stokes equation for incompressible flow is given by:

4 $$\uprho \, (\partial {\text{V}}/\partial {\text{t }} + {\text{ V }}\cdot\nabla {\text{V}}) \, = \, - \nabla {\text{P }} + \,\upmu \, (\nabla^{2} {\text{V}}) \, + \, \tau$$

where ρ is the fluid density, V is the velocity vector, t is time, ∇ is the gradient operator, P is the pressure, μ is the dynamic viscosity, ∇^2^ is the Laplacian operator, and τ is the deviatoric stress tensor. (b). Bingham Constitutive Equation: The Bingham constitutive equation relates the deviatoric stress tensor (τ) to the velocity gradient (∇V) in a Bingham fluid. It can be expressed as:

5 $$\tau \, = \, \tau_{0} + \,\upmu {\text{B }}(\nabla {\text{V}})$$

where τ_0_ is the yield stress, μB is the plastic viscosity, and (∇V) is the velocity gradient tensor. (c). Combining Equations: Substitute the Bingham constitutive equation into the conservation of momentum equation:

6 $$\uprho \, (\partial {\text{V}}/\partial {\text{t }} + {\text{ V }}\cdot\nabla {\text{V}}) \, = \, - \nabla {\text{P }} + \,\upmu \, (\nabla^{2} {\text{V}}) \, + \, \tau_{0} + \,\upmu {\text{B }}(\nabla {\text{V}})$$

(d). Boundary Conditions: Specify appropriate boundary conditions for the problem, such as velocity boundary conditions and pressure conditions. (e). Solve the Equations: Solve the coupled system of equations using numerical methods such as finite difference, finite element, or finite volume methods^22^. Various software packages and computational fluid dynamics (CFD) solvers are available that can handle such complex fluid flow problems^22^. It’s worth noting that solving the Bingham plastic model equations can be computationally demanding due to the presence of non-linearity and the need to handle the yield stress. Advanced numerical techniques and iterations may be required to obtain a solution. The Dirac delta function is a mathematical distribution that is often used to represent concentrated forces or point sources in fluid flow problems^22^. However, when it comes to solving equations for Bingham fluid flow, the presence of the Dirac delta function may not be directly applicable^11^. This is because the Bingham model assumes a continuous flow behavior, and the Dirac delta function represents an infinitely concentrated force or source^12^. Instead, in Bingham fluid flow, the focus is on solving the governing equations, such as the conservation of momentum (Navier–Stokes) equation and the Bingham constitutive equation, to determine the flow behavior and velocity distribution throughout the fluid domain^22^.

The v-funnel test is commonly used to assess the flowability or workability of self-compacting concrete (SCC). The test measures the time it takes for a given volume of concrete to flow through a standardized funnel^22^. The boundary conditions for v-funnel flowability of fresh SCC are influenced by various factors, including the aggregate characteristics and their heterogeneity^22^. However, the specific equations for the v-funnel test do not explicitly consider the heterogeneity of aggregates^1^. Instead, they focus on the flow behavior of the concrete mixture as a whole^22^. The v-funnel test is typically conducted according to a standardized procedure, such as ASTM C1611 or EN 12350-9. These standards define the apparatus and provide guidelines for performing the test. The test involves filling a v-funnel with a specific volume of concrete and measuring the time it takes for the concrete to flow through the funnel into a receiving container.

During the test, certain boundary conditions are imposed to ensure consistent and reproducible results^22^. These conditions include: (a). Funnel Geometry: The dimensions and shape of the v-funnel are specified in the standards. The internal diameter and height of the funnel, as well as the angle of its walls, are designed to create a specific flow pattern for the concrete^1^. (b). Sample Preparation: The concrete sample used in the test should be representative of the entire mixture^22^. It should be thoroughly mixed using appropriate mixing procedures for SCC^22^. The sample should also be free from any segregation or excessive bleeding. (c). Consistency: The concrete mixture should have a specific consistency, often specified using the slump flow test or similar tests^22^. The SCC should be able to flow freely through the funnel without any blockage or excessive resistance. (d). Test Environment: The v-funnel test should be conducted in a controlled environment at a specified temperature. The concrete sample and the funnel should be maintained at a consistent temperature throughout the test^22^. (e) Measurement: The time taken for the concrete to flow through the funnel is measured using a stopwatch or automated timing device. The test is typically repeated multiple times, and the average flow time is reported. While the equations used in the v-funnel test do not explicitly consider the heterogeneity of aggregates, the test results can indirectly reflect the influence of aggregate characteristics on the flowability of SCC. Aggregates with a wide range of particle sizes or significant variations in shape and surface texture can affect the flow behavior of the concrete mixture^22^. These variations may result in changes in the flow time or flow pattern observed during the v-funnel test. To account for the heterogeneity of aggregates in SCC, it is essential to carefully select and proportion the aggregate gradation, taking into consideration the desired flowability and the specific requirements of the project^22^. Additionally, optimizing the mix design and using appropriate admixtures can help enhance the flowability and mitigate the effects of aggregate heterogeneity. It is important to note that the v-funnel test is just one method for assessing the flowability of SCC. Other tests, such as the slump flow test, L-box test, or U-box test, may also be used in conjunction to provide a more comprehensive evaluation of the fresh concrete’s workability.

The v-funnel test measures the flowability of self-compacting concrete (SCC) using a standardized apparatus. The test does not have specific mathematical equations that directly consider the heterogeneity of aggregates. However, the test results can indirectly reflect the influence of aggregate characteristics on the flowability of SCC^22^. To understand the mathematics behind the v-funnel flowability test, we can look at the basic principles involved. The v-funnel test measures the time it takes for a specific volume of concrete to flow through a funnel. The flow time is influenced by the rheological properties of the concrete, including its viscosity, yield stress, and shear rate^6^. The flow time in the v-funnel test can be affected by the particle size distribution, shape, and surface texture of the aggregates^1^. Aggregates with a wide range of particle sizes or significant variations in shape can increase the internal friction within the concrete mixture, resulting in longer flow times^9^. Conversely, well-graded aggregates with smoother surfaces can enhance the flowability and reduce the flow time. While there are no specific equations that directly incorporate aggregate heterogeneity into the v-funnel test, there are mathematical models and empirical relationships that can help analyze the flow behavior of SCC^10^. These models consider the rheological properties of the concrete, such as its viscosity and yield stress, and can be used to predict flow times or simulate the flow behavior in different geometries^19^. One commonly used rheological model for SCC is the Bingham model^2^. The Bingham model describes the flow of a material with a yield stress and a linear relationship between the shear stress and shear rate^22,24^. This model can be used to analyze the flow behavior of SCC and estimate its flow time through the v-funnel^22^. However, it is important to note that the Bingham model and other rheological models do not explicitly consider the heterogeneity of aggregates^22^. They focus on the overall flow behavior of the concrete mixture rather than the specific influence of aggregate characteristics. To account for the heterogeneity of aggregates in SCC, a comprehensive approach is required^23^. This includes optimizing the aggregate gradation to minimize particle size variations, selecting aggregates with suitable shape and surface texture, and adjusting the mix design and admixtures to enhance flowability^24^. Additionally, empirical relationships and previous experience can provide guidance on the expected flow behavior based on the aggregate characteristics and their interaction with the fresh concrete mixture^20^. In summary, while there are no specific mathematical equations that directly incorporate aggregate heterogeneity into the v-funnel test, understanding the rheological properties of SCC and considering the influence of aggregate characteristics can help evaluate and optimize the flowability of fresh self-compacting concrete^23^. It is important to note that previous research works have tried to use different numerical methods to model the flowability of the SCC, which included the finite element method (FEM), the materials point method (MPM), discrete element method (DEM), and even the SPH^1,3,8,14^. These numerical methods except the SPH are based on the Eulerian algorithm. However, this present research work has tried to create a hybrid interface between the lagrangian-based meshless SPH algorithm and the ANSYS solver to take care of large deformation and avoiding mesh distortions. The earlier numerical methods experience mesh distortions leading to mathematical instabilities due to their inability to handle large deformation problems, but the hybrid interface created by the coupled SPH-ANSYS workspace handles this problem^1^(Supplementary Information 1).

## Results and discussion

### Model geometry and parametric overview

**Figure 2:**
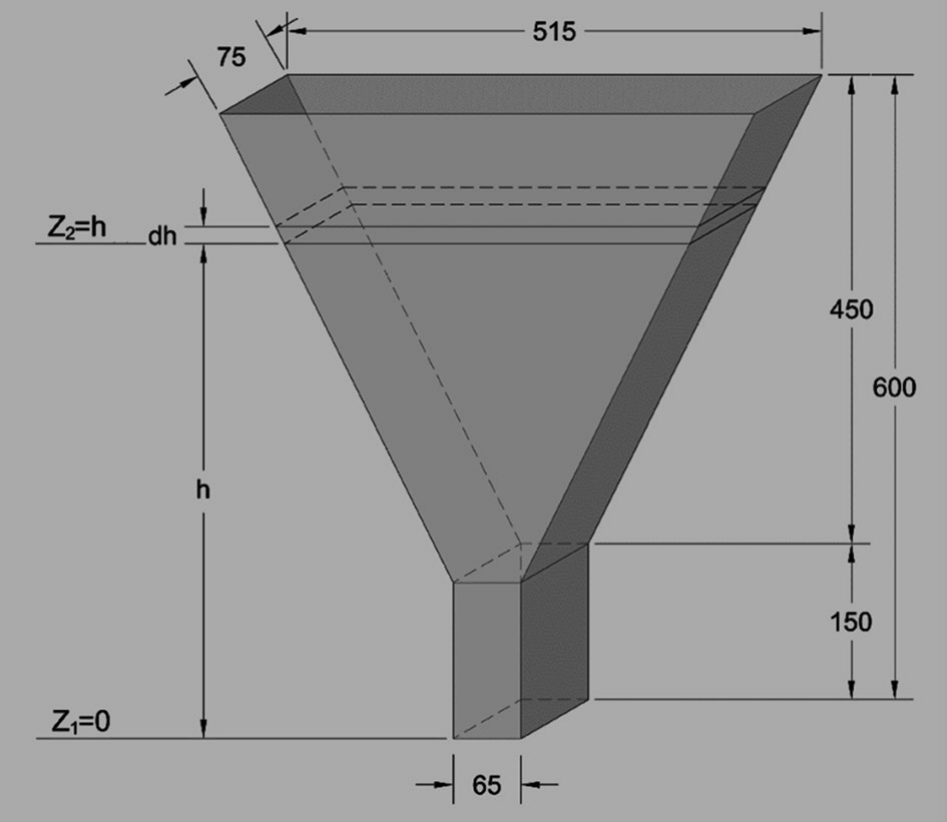
The V-funnel test method geometry.

Figure 2 shows the geometry of the modeled apparatus. Number of nonhomogeneous particles (coarse and fine) considered in each test setup = infinite, density/concrete unit weight = 2400 kg/m^3^, w/c ratio = 0.43, w/b ratio = 0.43, flow pattern = under gravity, fluid property = Bingham non-Newtonian (varying viscosity) incompressible fluid; Model Cases; 1: 0% coarse particles and 100% fine particles, 2: 60% coarse particles and 40% fine particles, 3: 55% coarse particles and 45% fine particles, 4: 50% coarse particles and 50% fine particles, 5: 45% coarse particles and 55% fine particles, 6: 40% coarse particles and 60% fine particles and 7: 100% coarse particles and 0% fine particles. These ratios are considered in line with allowable mix ratios provided by the EFNARC^22^ requirements for SCC production. The maximum size of the coarse aggregates is considered 20 mm and that of the fine aggregates is below 4 mm^22^. The Bingham model properties for Multiphysics (SPH)-ANSYS models’ simulation are; viscosity = 20 ≤ μ ≤ 100 and the yield stress = 50 $\le {\tau }_{0}\le 200$, standard flow time, t (s) ranges; 6 ≤ t ≤ 25 and apparatus volume is 12 L. These boundary conditions are provided by the EFNARC rheological requirements for the production of SCC^22^.

#### Multiphysics V-funnel flow optimization based on aggregate heterogeneity

**Figure 3:**
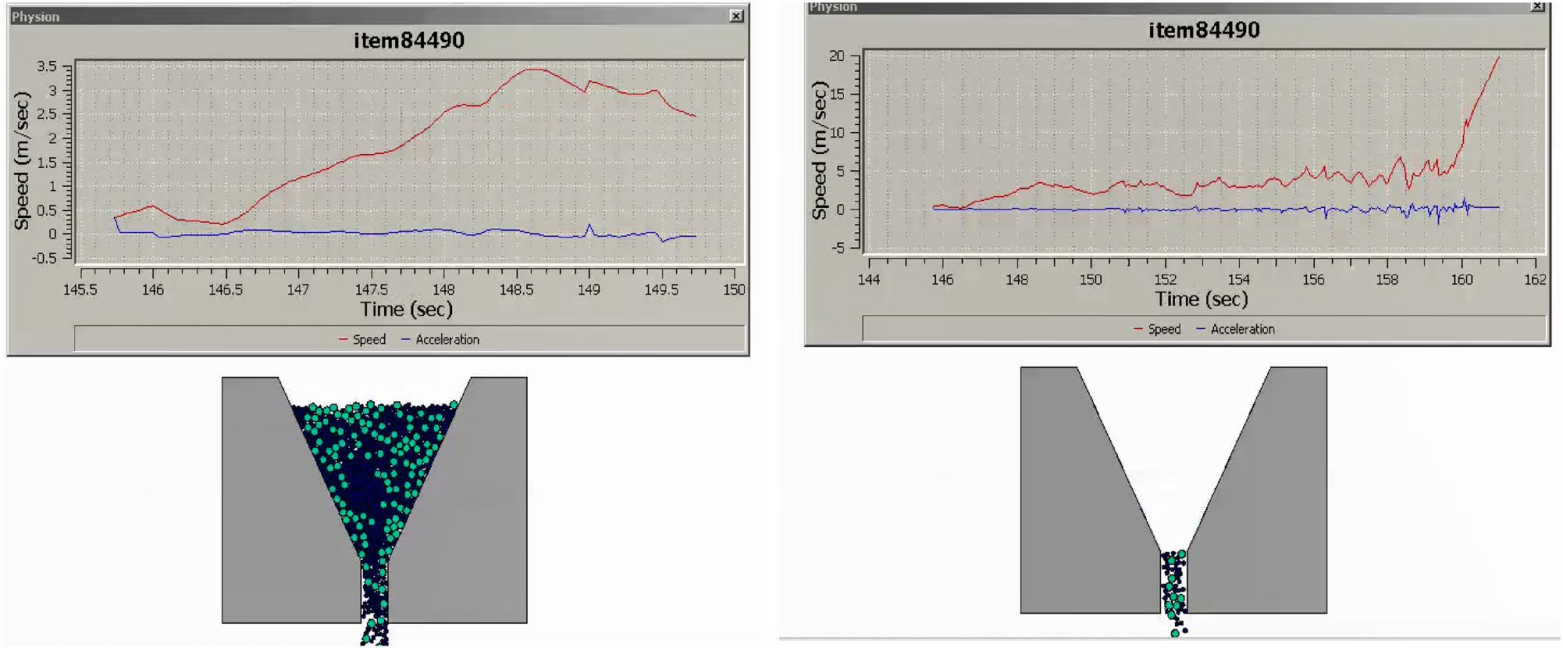
40%C mixed with 60%F case multiphysics model with V-funnel flow time (VFFT) of 16 s.

**Figure 4:**
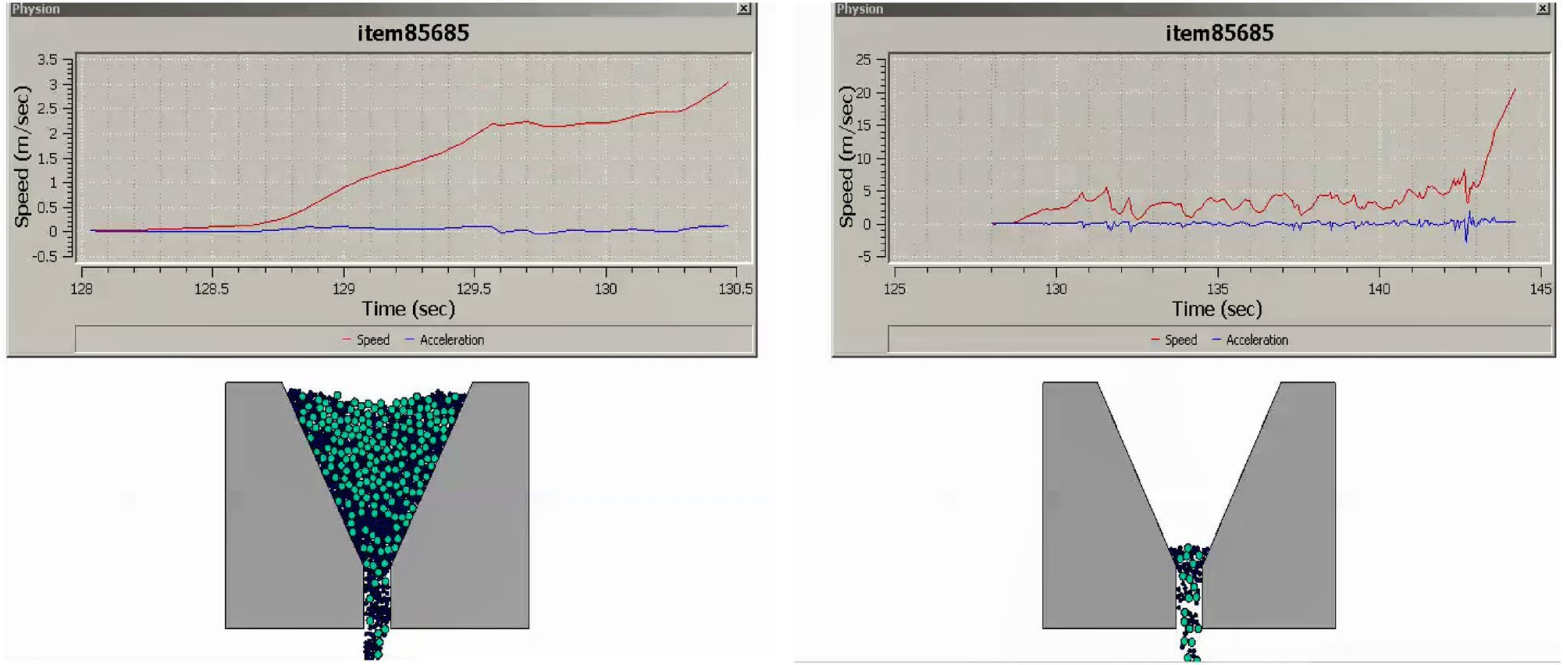
45%C mixed with 55%F case multiphysics model with V-funnel flow time (VFFT) of 17 s.

**Figure 5:**
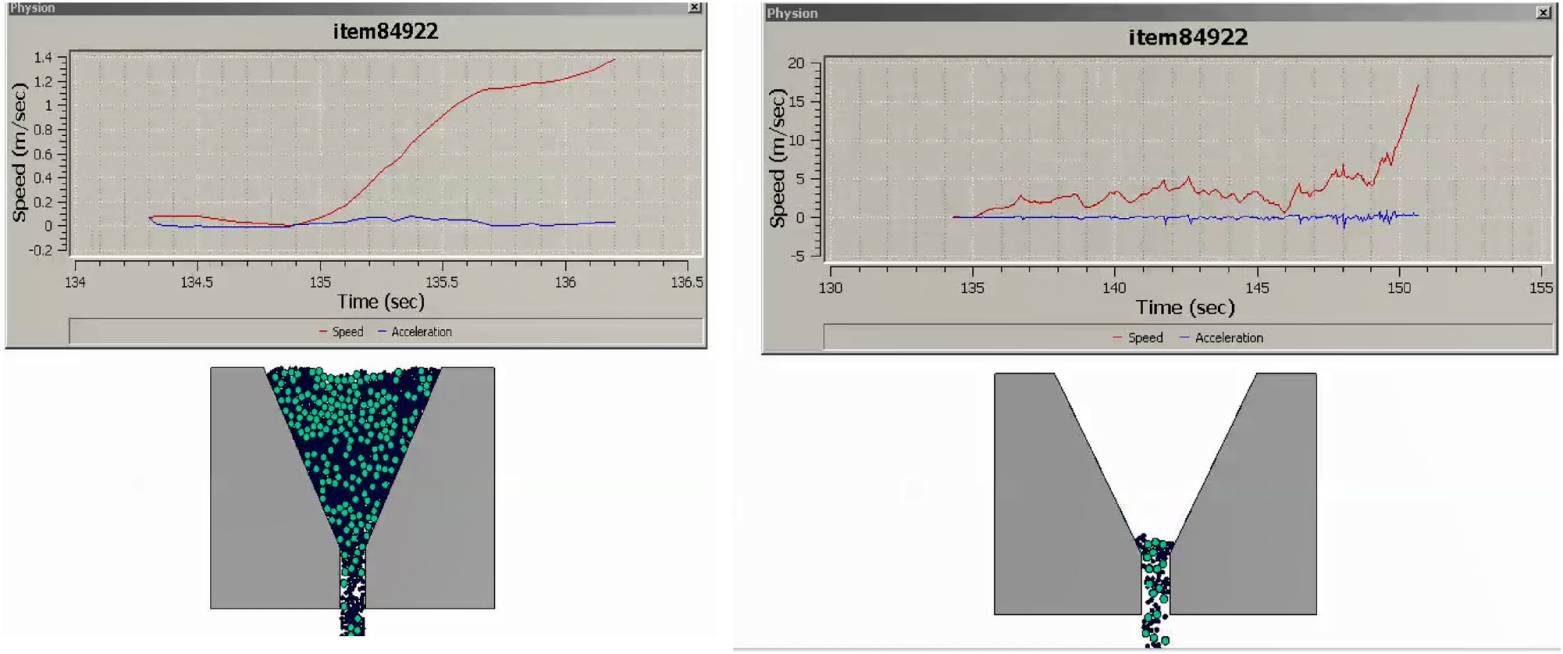
50%C mixed with 50%F case multiphysics model with V-funnel flow time (VFFT) of 18 s.

**Figure 6:**
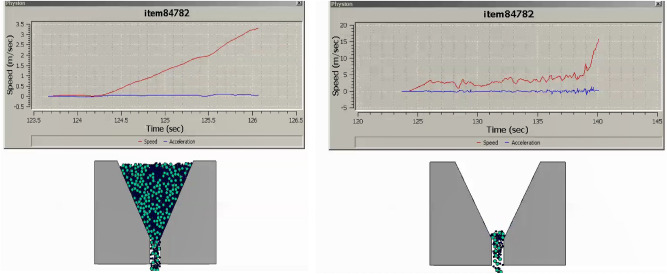
55%C mixed with 45%F case multiphysics model with V-funnel flow time (VFFT) of 18 s.

**Figure 7:**
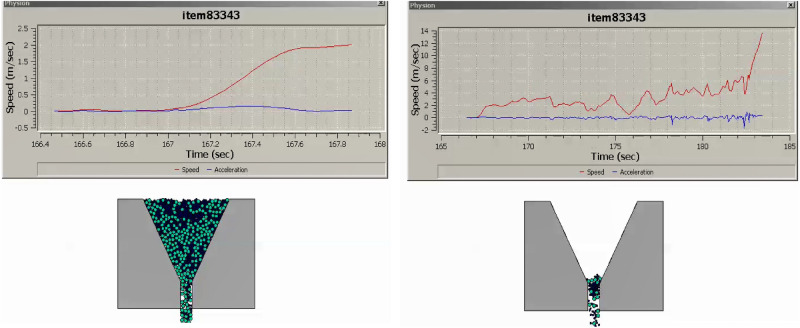
60%C mixed with 40%F case multiphysics model with V-funnel flow time (VFFT) of 18 s.

**Figure 8:**
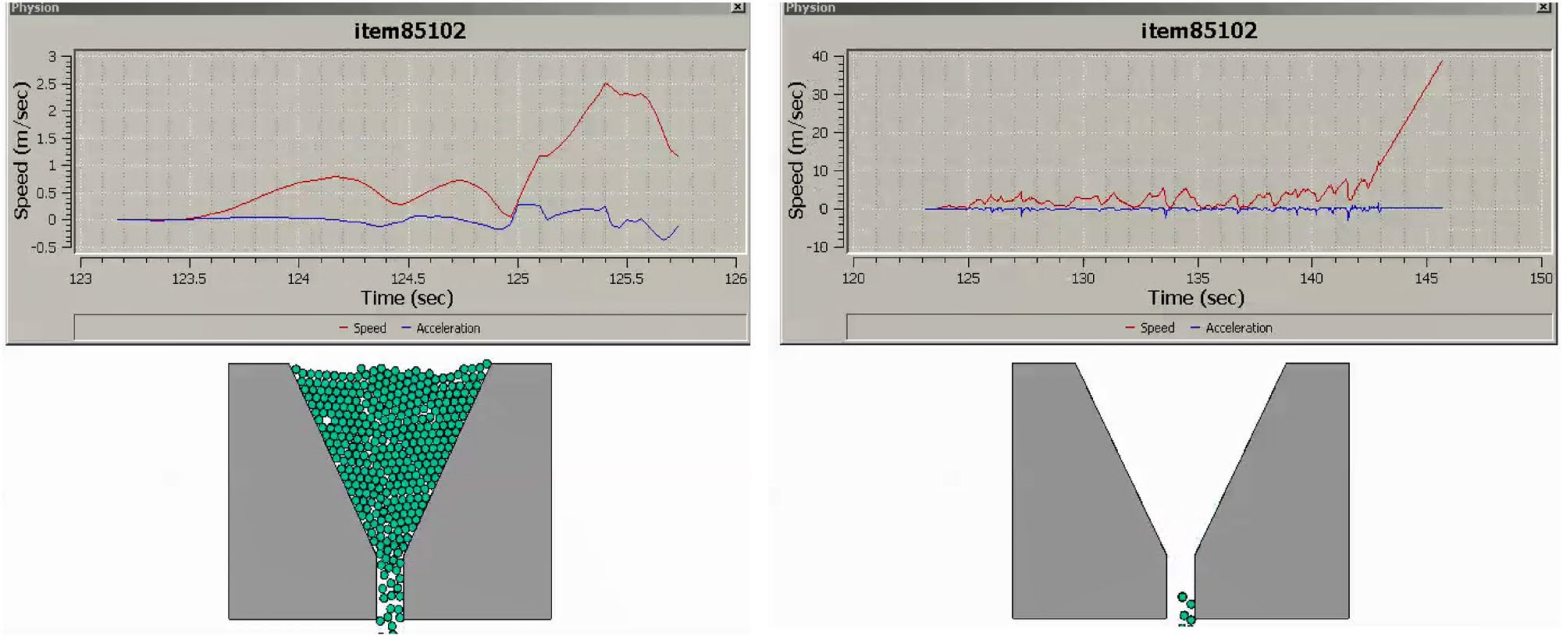
0%C mixed with 100%F case multiphysics model with V-funnel flow time (VFFT) of 14 s.

The research undertaken in this study focuses on modeling and optimizing the apparatus filling ability of self-compacting concrete (SCC). This characteristic pertains to SCC’s capacity to flow and fill formwork or molds without the need for mechanical compaction, and it has been approached in line with the mix percentages of coarse and fine aggregates following EFNARC guidelines^22^. SCC is engineered for high flowability and viscosity, enabling smooth passage through congested reinforcement, ensuring complete formwork filling under proportionate heterogeneity sampling of aggregates. Previous considerations in this domain encompassed statistical methods and the use of recycled aggregate replacements for normal aggregates^23–25^, adopting suitable research methodologies^26^. To analyze the modeled filling ability of SCC, several crucial factors have been taken into account: (i) Viscosity: The SCC’s viscosity adheres to the boundary condition of 20 ≤ μ ≤ 100^22^ to ensure optimal filling ability. Viscosity can be measured through methods like the V-funnel test or the U-box test, with lower viscosity facilitating better flow and formwork filling. (ii) Segregation Resistance: SCC should resist segregation, evaluated through visual inspection and aggregate segregation tests to prevent poor filling and reduced homogeneity. (iii) Passing Ability: SCC must pass through congested reinforcement without blockage or excessive pressure to fill complex formwork configurations effectively. (iv) Rheological Properties: Rheological properties, including yield stress, plastic viscosity, and thixotropy, are considered in line with the Bingham plastic model. These properties can be measured using rheometers or viscometers. The minimum v-funnel flow time, a critical parameter in analyzing SCC’s filling ability, has been modeled for seven different scenarios (model cases) (see supplementary file) involving varying proportions of coarse and fine aggregates as provided by EFNARC. Each case represents the time (6 ≤ t ≤ 25 s) it takes for SCC to completely flow through a specified distance. The obtained results for the seven cases are presented in Figs. 3, 4, 5, 6, 7 and 8, showcasing the start and end of the flow time. The minimum flow time serves as an indicator of SCC’s ability to rapidly fill formwork or molds without additional compaction. To further analyze the filling ability based on minimum flow time, the following steps have been taken: (i) Measurement and Analysis: The timing starts when SCC initiates flow and stops when the flow ceases. The recorded time in seconds represents the minimum flow time. (ii) Interpretation: The minimum flow time provides insights into SCC’s filling ability. A shorter minimum flow time suggests better flowability and faster filling, indicating SCC’s capability to fill formwork without additional compaction. (iii) Comparison and Optimization: Compare the obtained minimum flow time with specifications for the specific application. Adjustments to the mix design, including modifications to aggregate grading, water content, or the use of chemical admixtures, can be made to improve SCC’s filling ability. Comparing the seven model cases, it is evident that the flow time has been optimized in the scenario where 40% coarse aggregates are mixed with 60% fine aggregates, resulting in a flow time of 16 s. This optimization aligns with EFNARC guidelines, which specify 6–25 s as the standard flow time through the studied and modeled apparatus. It’s crucial to note that the minimum flow time has been considered alongside other relevant parameters and tests, such as slump flow, passing ability, segregation resistance, and rheological properties, to comprehensively assess SCC’s filling ability in this model. In conclusion, the proportion of coarse and fine aggregates significantly influences SCC’s filling ability and flow time. The specific effects of a 40% coarse aggregate and 60% fine aggregates mix on SCC’s filling ability depend on factors such as aggregate properties, mix design, and desired flowability. Potential influences include improved flowability, viscosity, and segregation resistance, enhanced packing density, and favorable workability retention. It is emphasized that conducting laboratory tests using actual materials and mix designs will provide a more detailed understanding of SCC’s filling ability and flow time in this particular scenario.

### ANSYS-SPH interface simulation of optimized V-funnel flow

**Figure 9:**
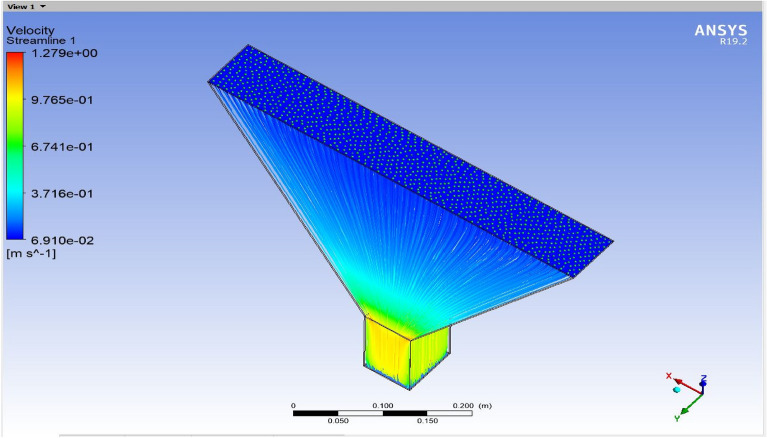
Funnel flow velocity and energy properties for the optimized mix.

**Figure 10:**
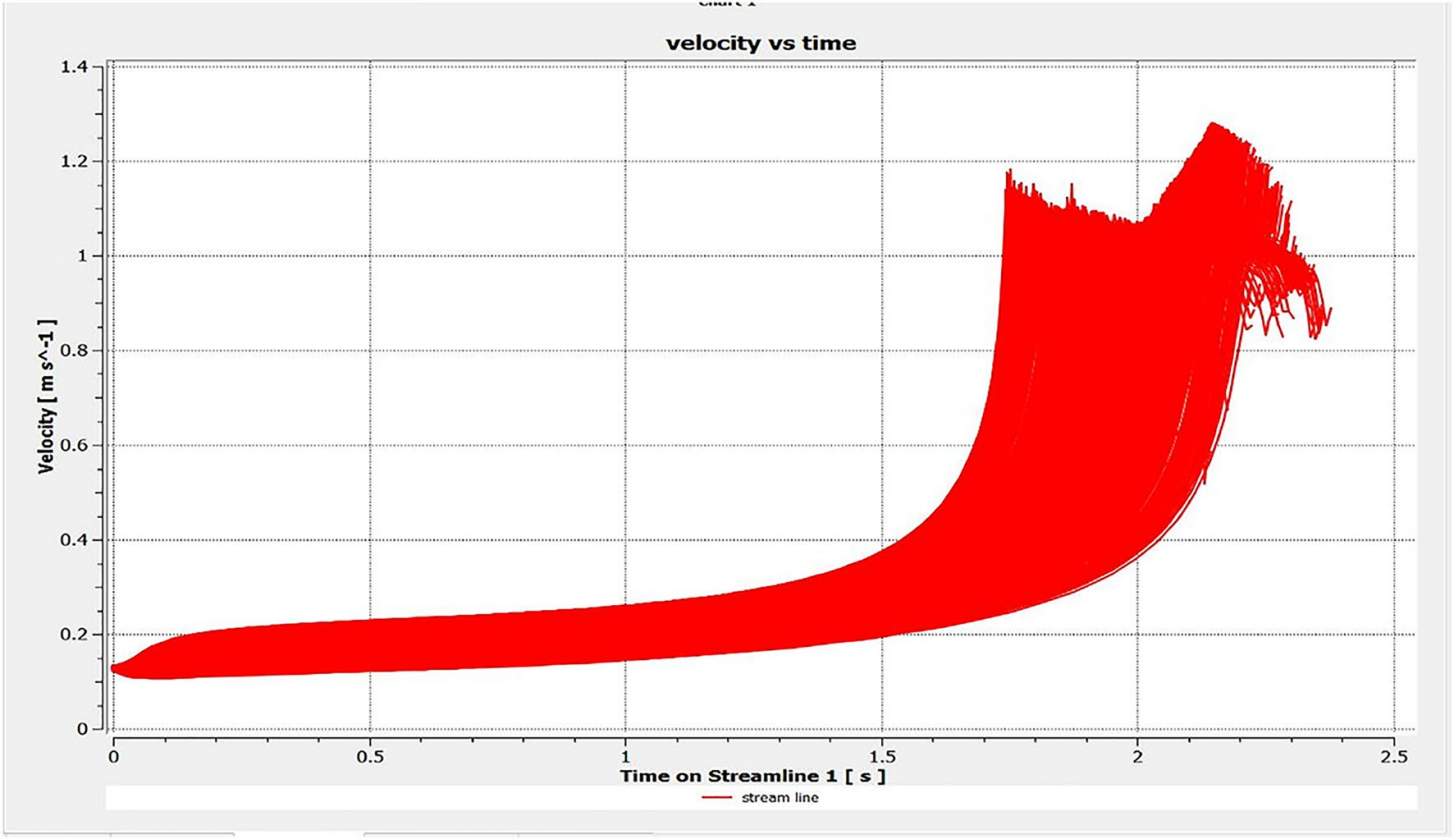
Flow velocity interparticle properties on the wall.

**Figure 11:**
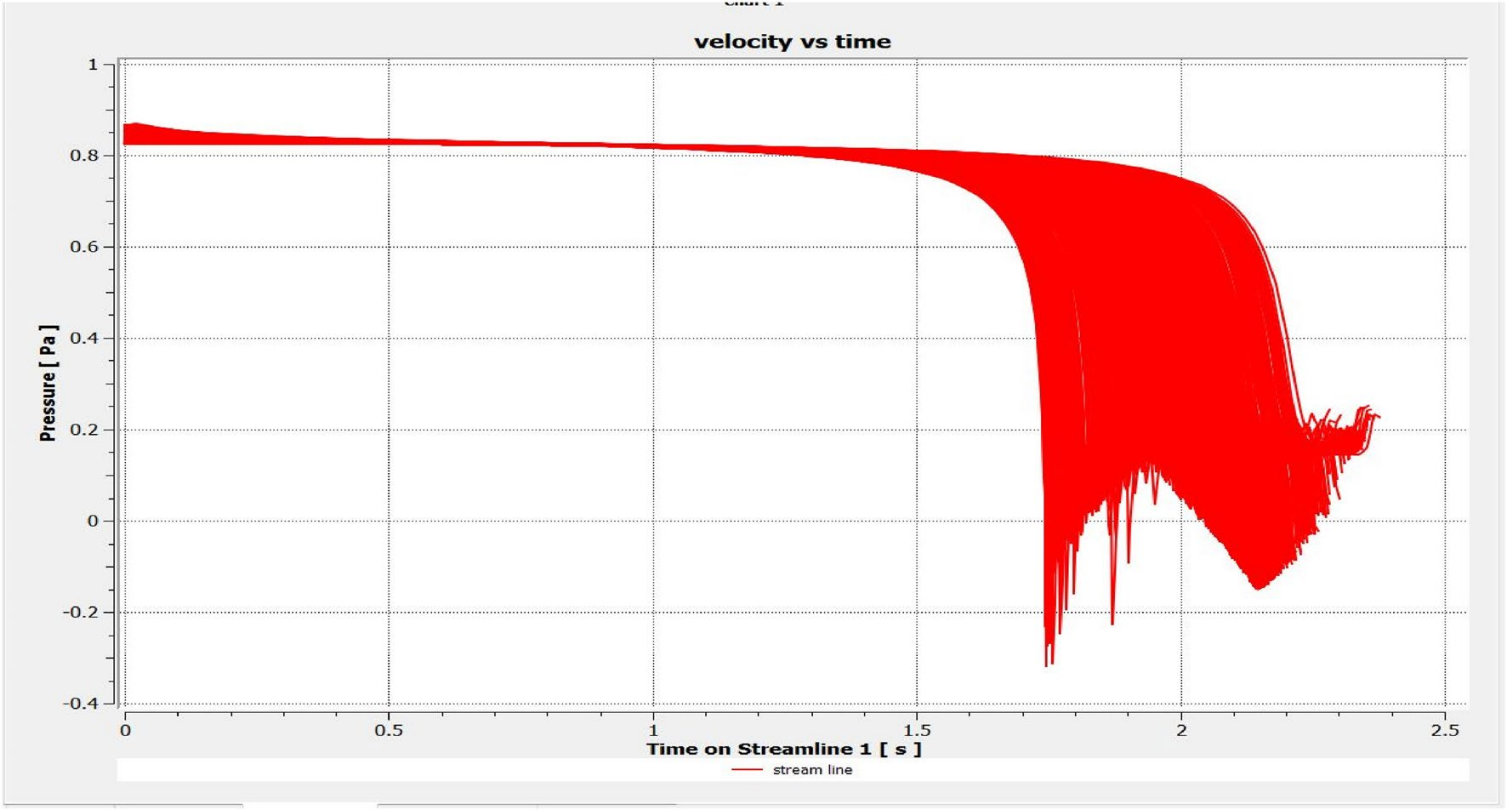
Flow pressure interparticle properties on the wall.

**Figure 12:**
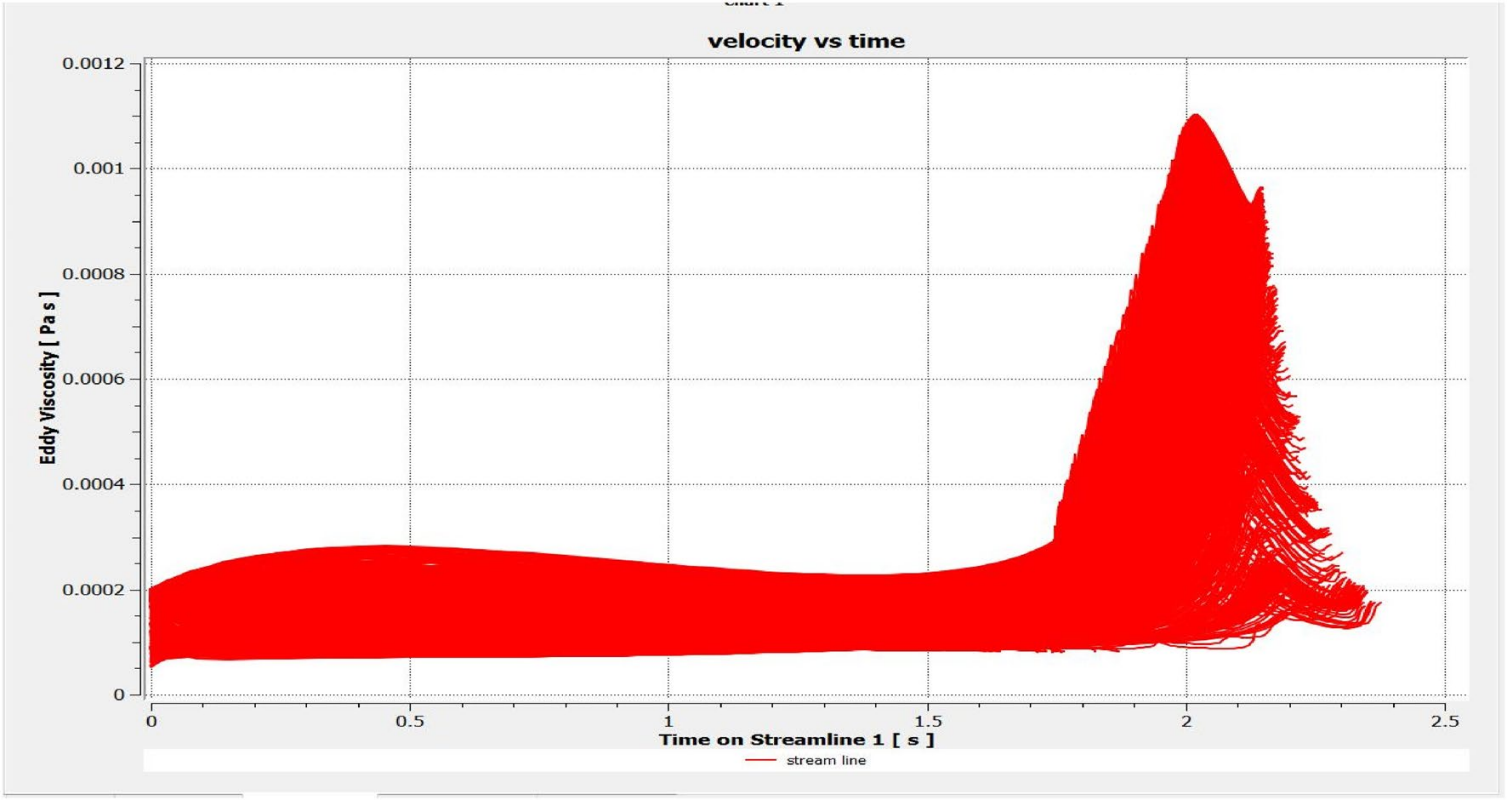
Flow Eddy viscosity interparticle properties on the wall.

**Figure 13:**
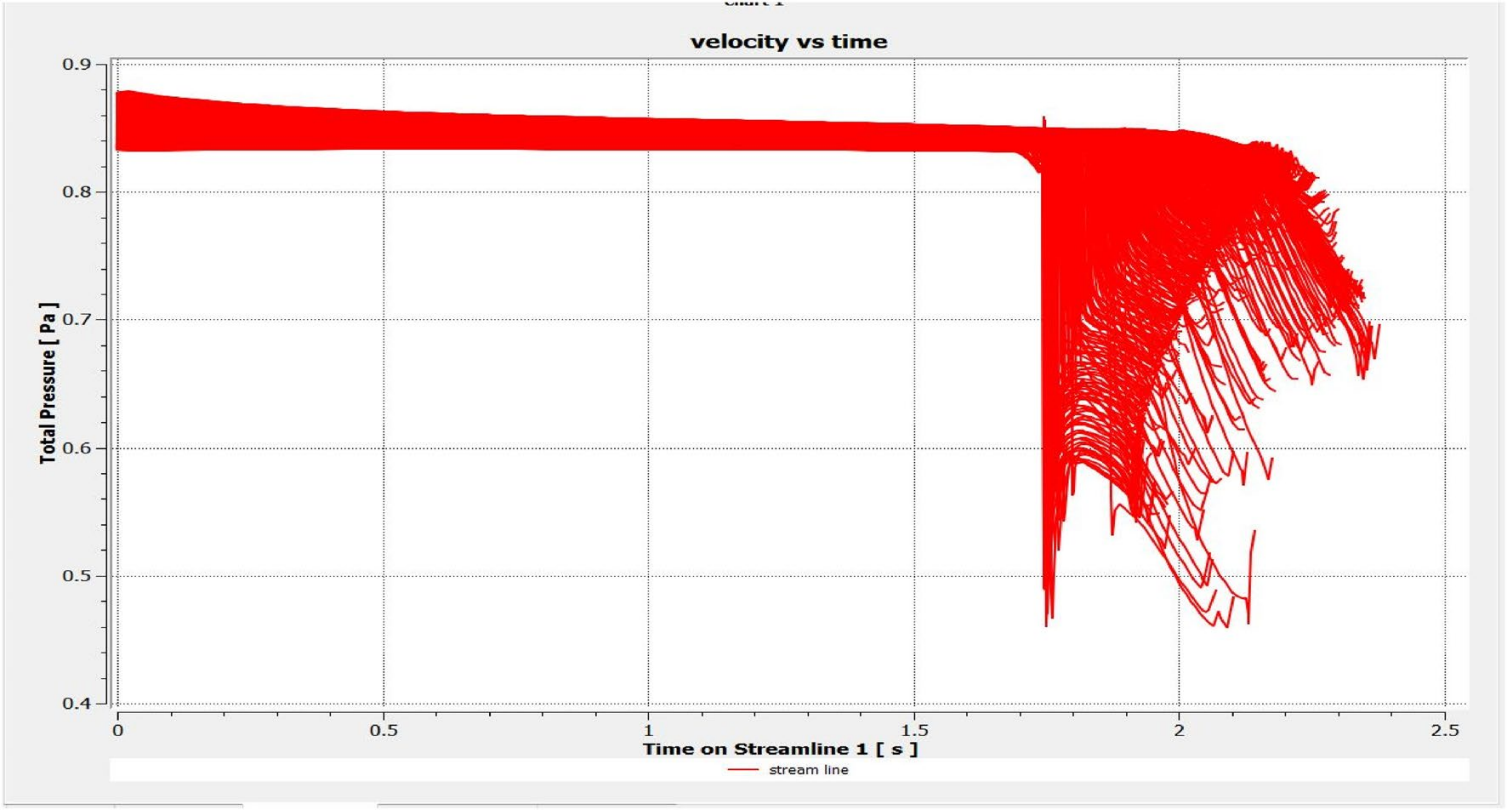
Flow total pressure interparticle properties on the wall.

**Figure 14:**
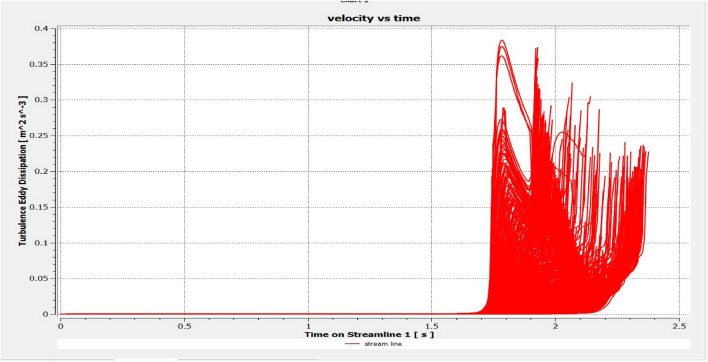
Flow turbulence Eddy dissipation interparticle properties on the wall.

**Figure 15:**
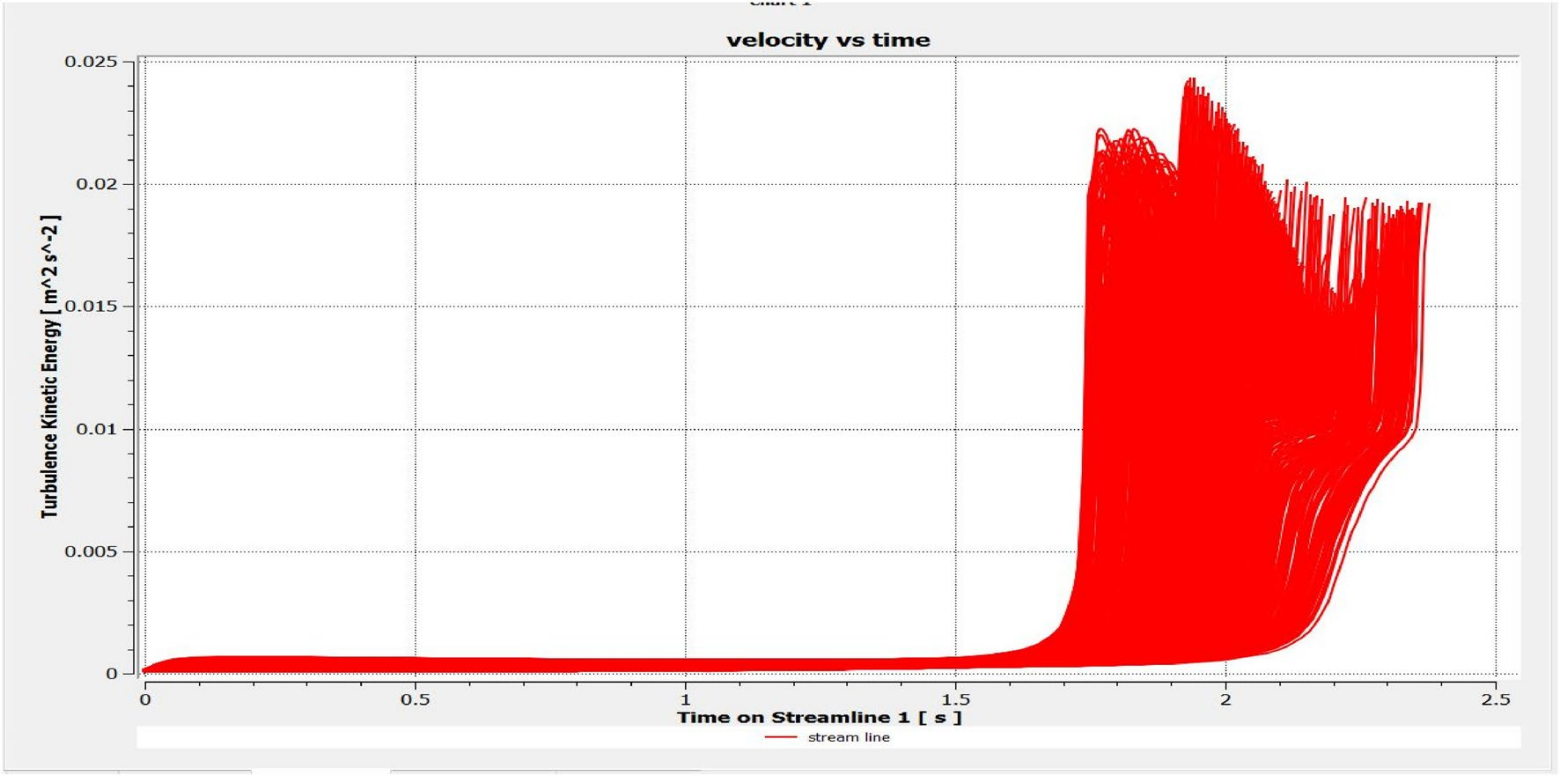
Flow turbulence kinetic energy interparticle properties on the wall.

**Figure 16:**
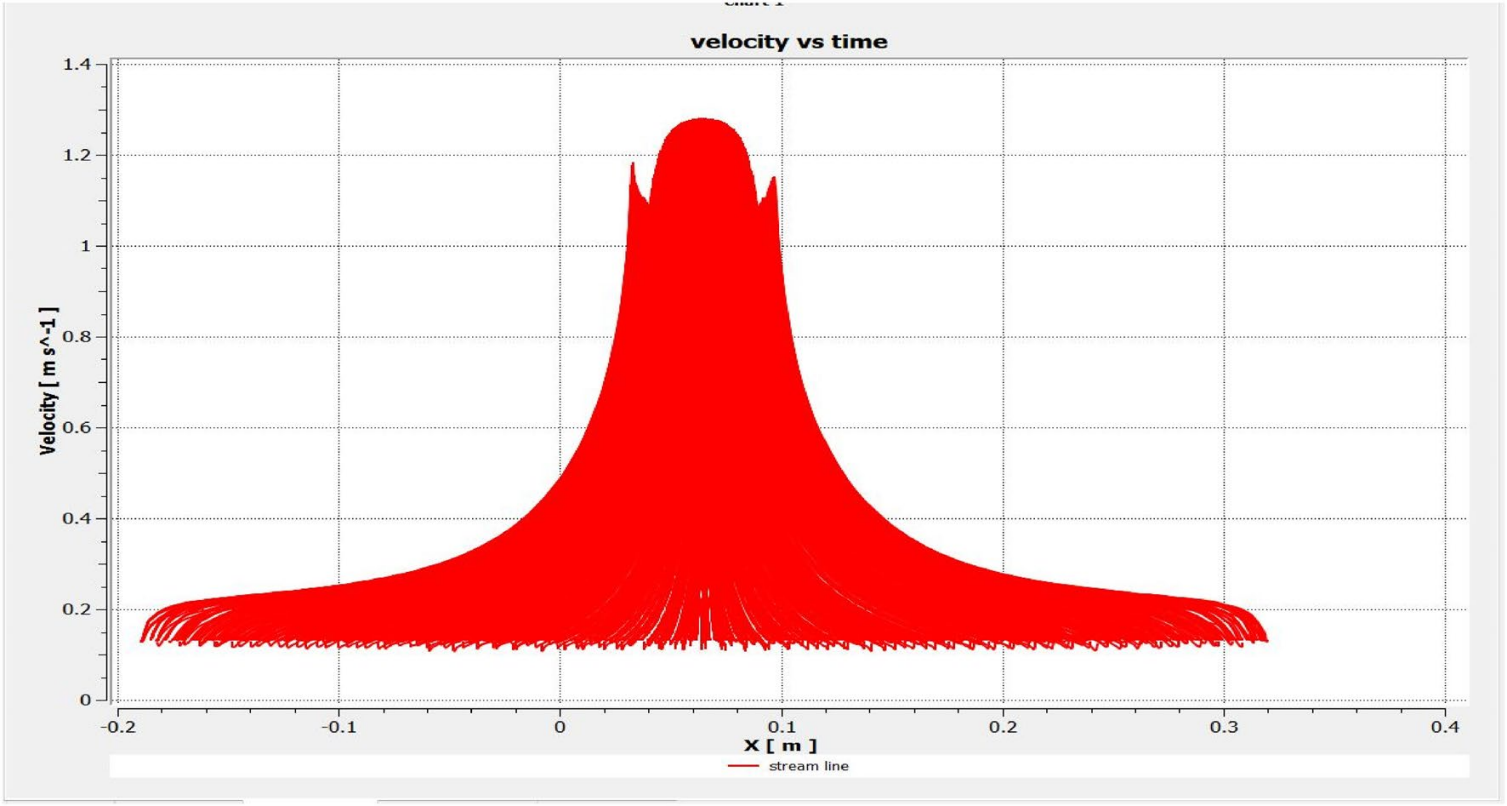
Flow filling velocity interparticle properties on the wall.

**Figure 17:**
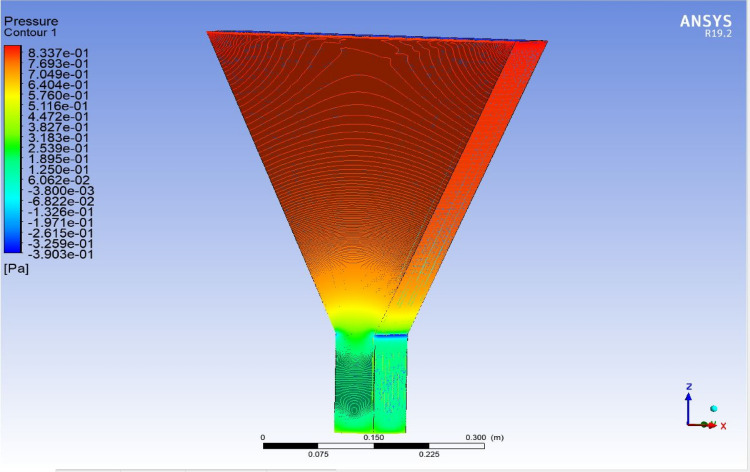
Pressure contour properties for the optimized mix.

**Figure 18:**
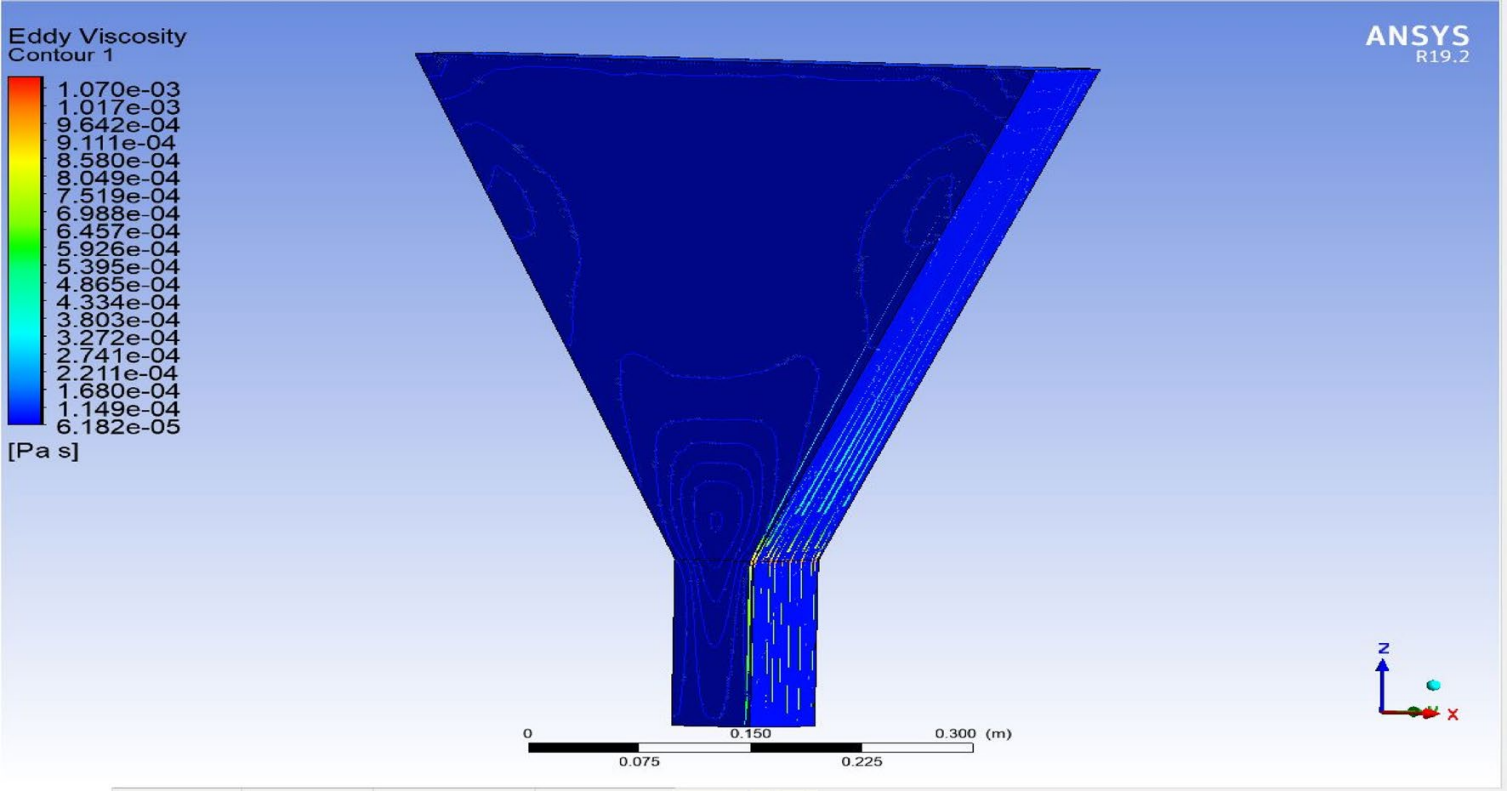
Eddy viscosity contour properties for the optimized mix.

**Figure 19:**
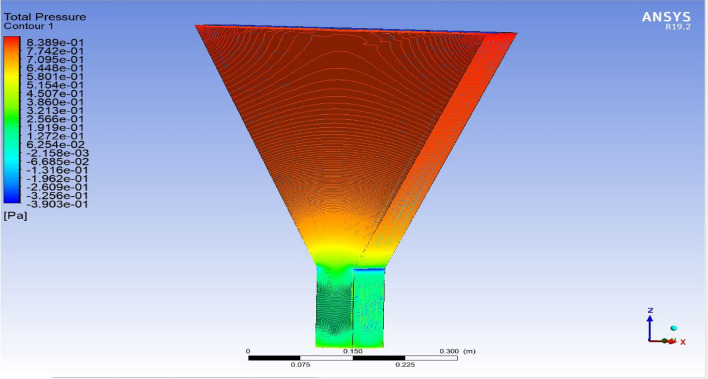
Total pressure contour properties for the optimized mix.

**Figure 20:**
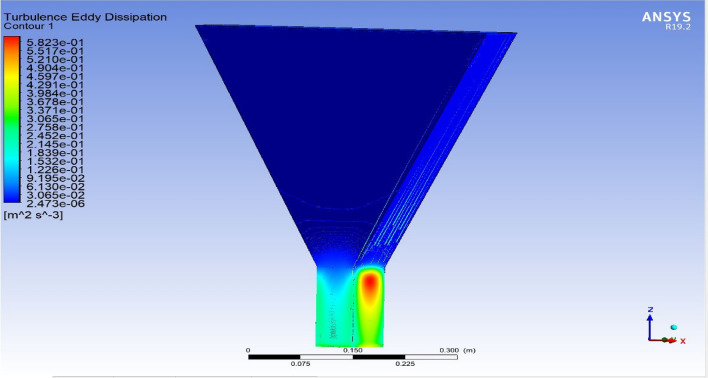
Turbulence Eddy dissipation contour properties for the optimized mix.

**Figure 21:**
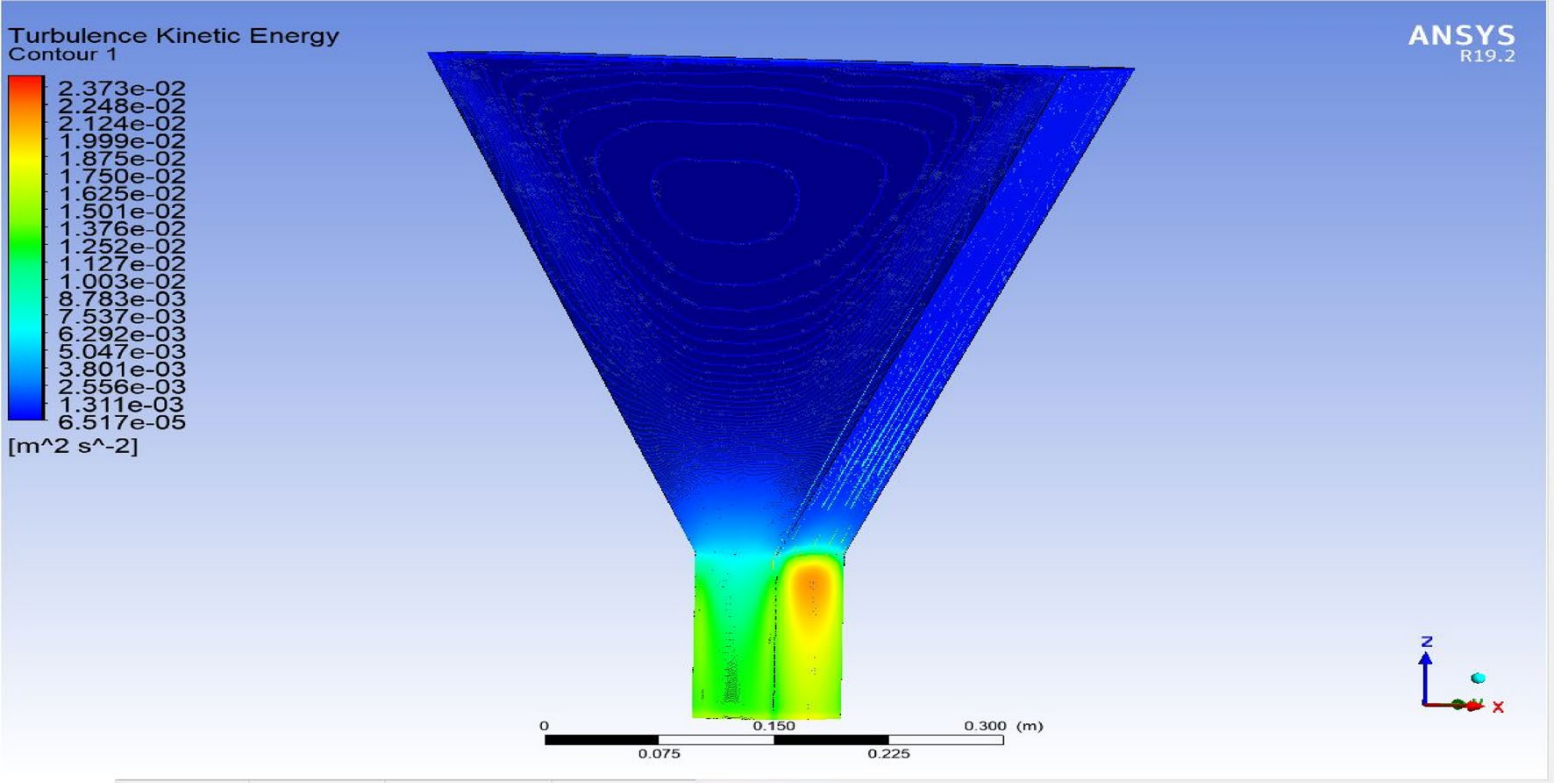
Turbulence kinetic energy contour properties for the optimized mix.

**Figure 22:**
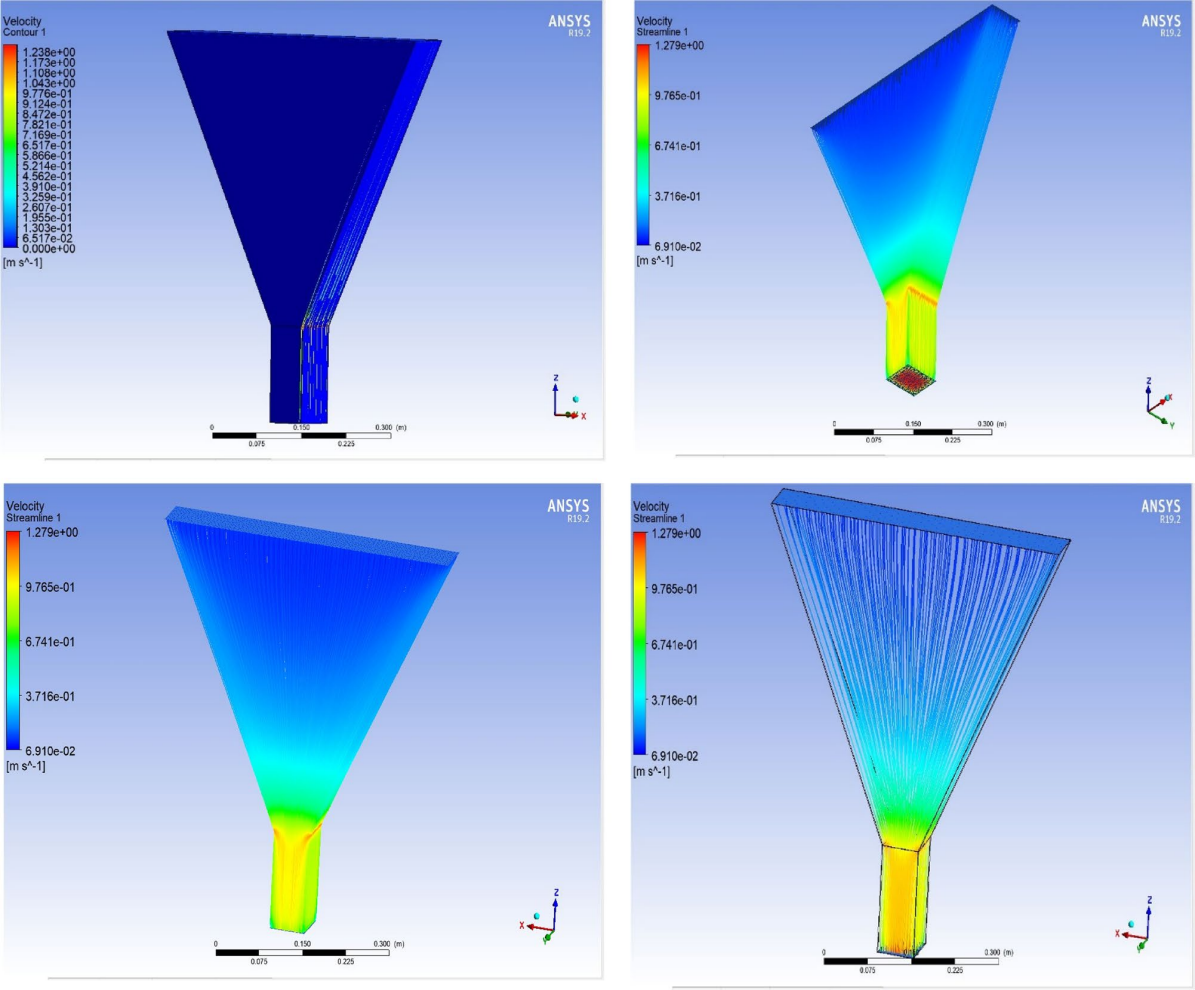
Velocity contour properties for the optimized mix.

**Figure 23:**
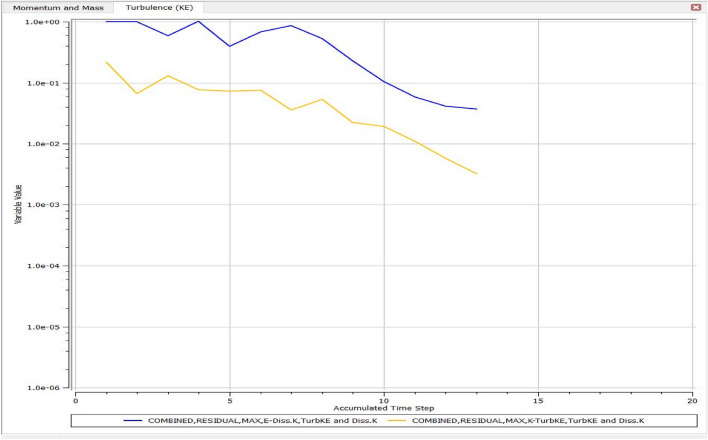
Turbulence in terms of the kinetic energy of the optimized SCC mix over accumulated flow time.

**Figure 24:**
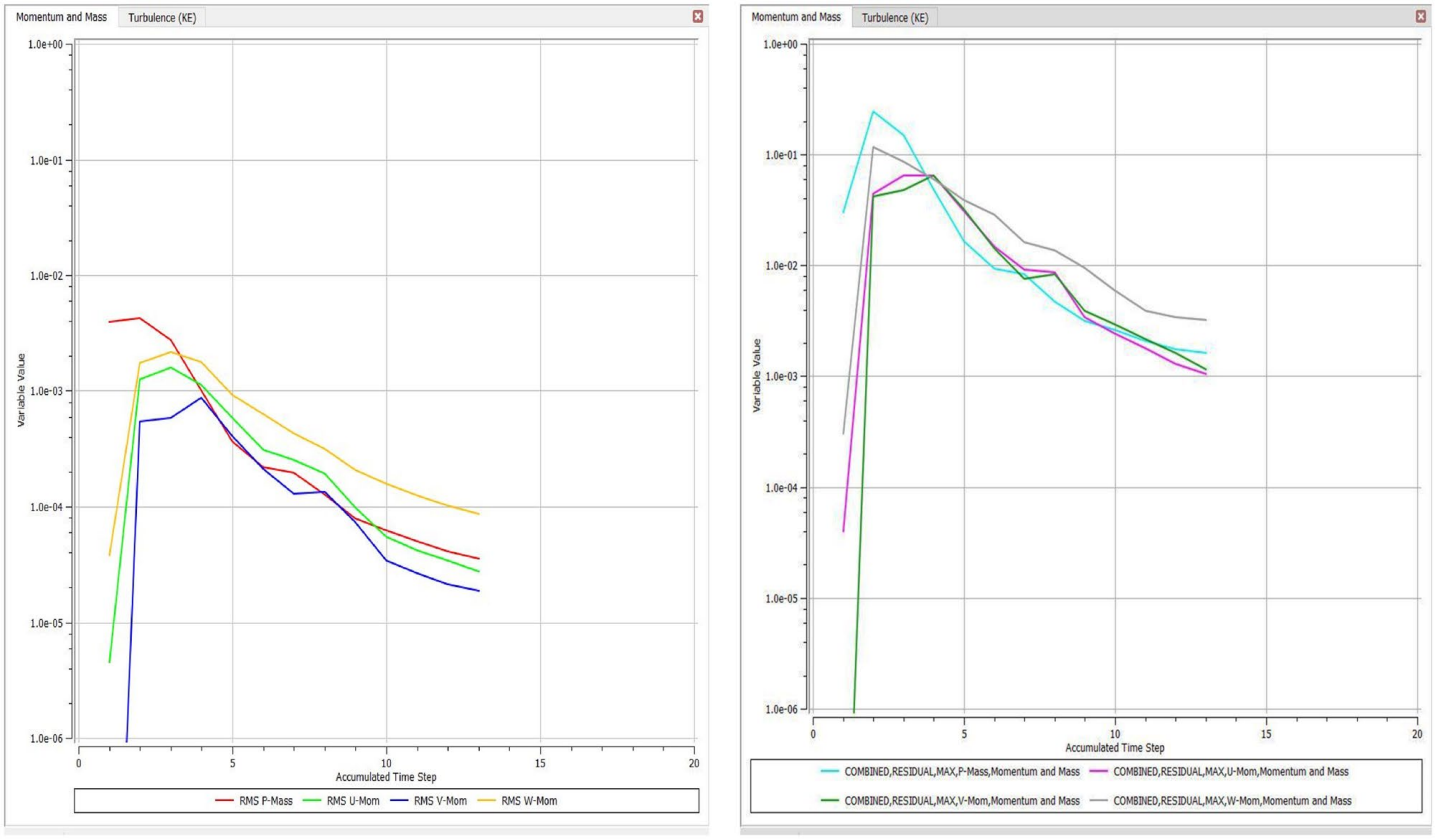
Momentum-mass relationship in terms of the kinetic energy of the optimized SCC mix over accumulated flow time.

The optimized multiphase mix (40%C + 60%F:16s) underwent further simulation using the coupled interface of the ANSYS-SPH platform, operating with the CFX command at an air temperature of 25 °C. This simulation incorporated a studied density of 2400 kg/m^3^, plastic viscosity boundary, yield stress, and aggregate sampling. The analytical space configuration of the smoothed particle hydrodynamics embedded in the fluid dynamic manipulation of the ANSYS solver was employed for this purpose. The model simulation involved a total number of nodes (453,220), total number of elements (409,032), total number of prisms (12,784), total number of hexahedrons (396,248), and total number of faces (100,126). The simulation results included Dynamic Viscosity (1.8310E−05 kg m^−1^ s^−1^), Thermal Conductivity (2.61E−02 W m^−1^ K^−1^), Absorption Coefficient (0.01 m^−1^), Refractive Index (1.0 m m^−1^), Molar Mass (28.96 kg kmol^−1^), Specific Heat Capacity (1.0044E + 03 J kg^−1^ K^−1^), Thermal Expansivity (0.003356 k^−1^), Normal Speed (132 mm s^−1^), Pressure Profile Blend (0.05), and Maximum Partition Smoothing Sweeps (100). Additionally, global parameters such as Global Length (2.1911E−01), Minimum Extent (7.5000E−02), Maximum Extent (6.0000E−01), Density (1.1850E + 00 kg/m^3^), Velocity (3.9130E−01 m/s), Advection Time (5.5994E−01), and Reynolds Number (5.5488E + 03) were part of the simulation. To simulate the optimized flow time and filling ability of a Self-Compacting Concrete (SCC) mixture with 40% coarse aggregate and 60% fine aggregate, 100 iterations and a coupled interface tool specifically designed for concrete mix design and simulation was employed. This tool takes into consideration various factors, including aggregate properties, water-cement ratio, admixtures, and other parameters, to predict the flow time and filling ability of the concrete mix. Simulation results, presented in Figs. 9, 10, 11, 12, 13, 14, 15, and 16, include the discretization of the funnel model apparatus, simulated velocity, pressure, total pressure dissipation, turbulence kinetic energy, and velocity streamline. The graphical representation of these simulated funnel flow characteristics at 16 s of optimized flow time are presented in Figs. 17, 18, 19, 20, 21, 22, 23, and 24. The simulation also provided data on wall forces and moments on the funnel wall for the optimized mix of 40%C + 60%F@16s as follows: Pressure force on funnel wall: x-component: 1.5926E−07, y-component: − 9.0399E−07, z-component: − 2.5116E−02. Viscous force on funnel wall: x-component: − 1.9588E−07, y-component: 1.0738E−08, z-component: − 3.5947E−04. Pressure moment on funnel wall: x-component: − 2.1167E−03, y-component: 1.6307E-03, z-component: − 1.3673E−07. Viscous moment on funnel wall: x-component: − 3.0295E−05, y-component: 2.3341E−05, z-component: 2.5233E−08. Additionally, the maximum residuals were identified at node 401,131 for pressure, node 441,245 for K-TurbKE, and node 451,431 for E-Diss.K. In the context of Self-Compacting Concrete (SCC) filling ability, the significance of Dynamic Viscosity (DV), Thermal Conductivity (TC), and Reynolds Number (RN) lies in their influence on the flow and behavior of the concrete mix. Dynamic viscosity represents the internal friction of the concrete mix, indicating its resistance to flow. In the modeled SCC, the lower dynamic viscosity is desirable as it allows the mix to flow more easily to achieve the desired standard flow time. Also, the lower dynamic viscosity promotes better flowability, enabling the SCC to navigate through complex formwork, reinforcement, and other structural elements without the need for additional compaction or vibration. The thermal conductivity measures the ability of the concrete mix to conduct heat. In the context of SCC also, it can influence temperature-related effects during handling/placement and curing. While thermal conductivity is not a direct factor in filling ability, it can indirectly affect SCC flow behavior due to hydration reaction. Higher thermal conductivity may influence the curing process and temperature distribution, which can, in turn, impact the rheological properties of the mix and its ability to flow and fill. And the Reynolds Number (RN) is a dimensionless quantity used to predict the flow patterns in fluid dynamics. It relates inertial forces to viscous forces and helps identify the flow regime. In this SCC case, a suitable Reynolds Number ensures that the flow remains within a predictable range^22^. Understanding the Reynolds Number is crucial to preventing issues like segregation or blockages in the flow, ensuring that the SCC maintains its desired filling ability. In summary, these parameters play roles in governing the rheological properties, heat transfer characteristics, and flow behavior of SCC. Optimizing dynamic viscosity, thermal conductivity, and Reynolds Number contributes to achieving the desired filling ability, ensuring that the SCC can efficiently fill complex molds and formwork without compromising its structural integrity.

## Conclusions

In this study, seven model cases were considered to investigate the influence of proportionate heterogeneity of aggregates on the v-funnel flow time of Self-Compacting Concrete (SCC). The cases included variations in the percentages of coarse and fine particles. The Bingham model properties for the Multiphysics (SPH)-ANSYS models’ simulation were set from the EFNARC requirements as follows: viscosity = 20 ≤ μ ≤ 100, yield stress = 50 ≤ τ_0_ ≤ 200, and standard flow time, t (s), ranged from 6 ≤ t ≤ 25. The apparatus volume of 12 L was considered. The minimum boundary flow time, representing the time for SCC to completely flow through a specified distance, was modeled for the seven cases. The results showed varying flow times, with the 0%C mixed with 100%F case flowing out completely in 14 s, while the 40%C mixed with 60%F case took 16 s, and so on. The minimum flow time was considered for the 40–60 mix alongside parameters such as slump flow, passing ability, segregation resistance, and rheological properties to comprehensively assess the filling ability of SCC. The optimized mix (40%C + 60%F:16s) was highlighted as a potential solution for achieving desired flowability and filling performance. The multiphase optimized mix (40%C + 60%F:16s) underwent further simulation using the coupled interface of the ANSYS-SPH platform. The simulation included parameters such as density, plastic viscosity boundary, yield stress, and aggregate sampling. The results produced dynamic viscosity, thermal conductivity, absorption coefficient, refractive index, molar mass, specific heat capacity, normal speed, pressure profile blend, and maximum partition smoothing sweeps. Wall forces and moments on the funnel wall for the optimized mix were also obtained, with maximum residuals identified at specific nodes for pressure. The study concludes by suggesting that future research could extend this model work to include other filling ability flow apparatuses. Generally, future research works should focus on the modeling the behavior of coarse aggregates greater than 20 mm as to study the deformation mechanism and the energy jump during the flow.

### Supplementary Information

Supplementary Information.

## Data Availability

The data supporting this research work is available on reasonable request from the corresponding author.
